# Supplementary material for: Antibody-assisted selective isolation of Purkinje cell nuclei from mouse cerebellar tissue
Source: Cell Rep Methods. 2024 Jul 8;4(7):100816. doi: 10.1016/j.crmeth.2024.100816 (PMC11294835; doi:10.1016/j.crmeth.2024.100816)
Supplement: Document S1. Figures S1–S4 and Table S1 [file mmc1.pdf]

Cell Reports Methods, Volume *4*

Supplemental information

Antibody-assisted selective isolation of Purkinje cell nuclei from mouse cerebellar tissue

Luke C. Bartelt, Mouad Fakhri, Grazyna Adamek, Magdalena Trybus, Anna Samelak-Czajka, Paulina Jackowiak, Agnieszka Fiszer, Craig B. Lowe, Albert R. La Spada, and Pawel M. Switonski

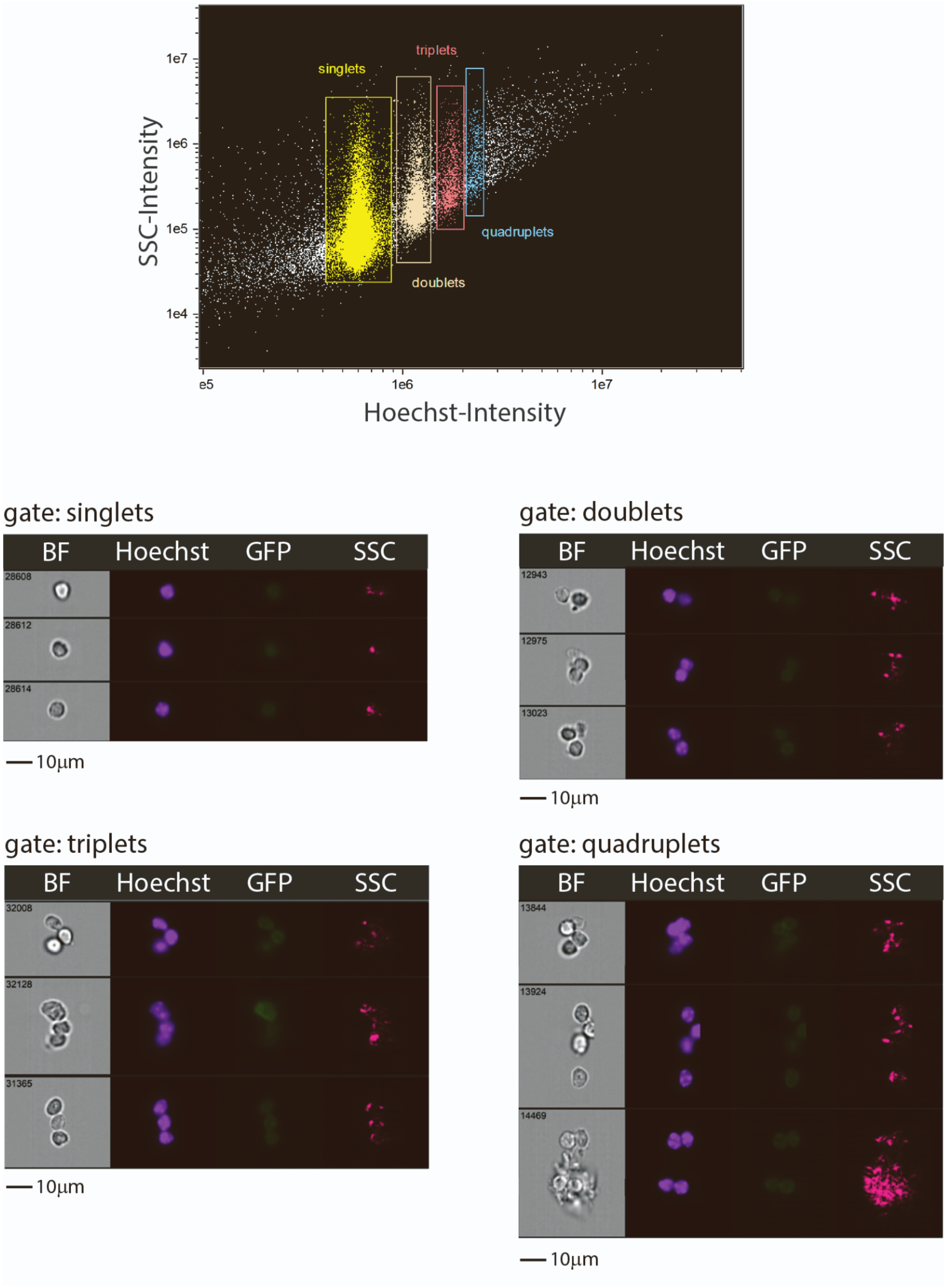

Supplementary Figure 1. Visual inspection of Hoechst staining patterns conducted on nuclei isolated from mouse cerebella, related to Figure 1.

Each cluster represents a gradual aggregation of nuclei, as verified through image cytometry. Representative examples are shown; Scale bar: 10µm. It is important to note that all nuclei sorted and analyzed in this study were selected from the singlets gate.

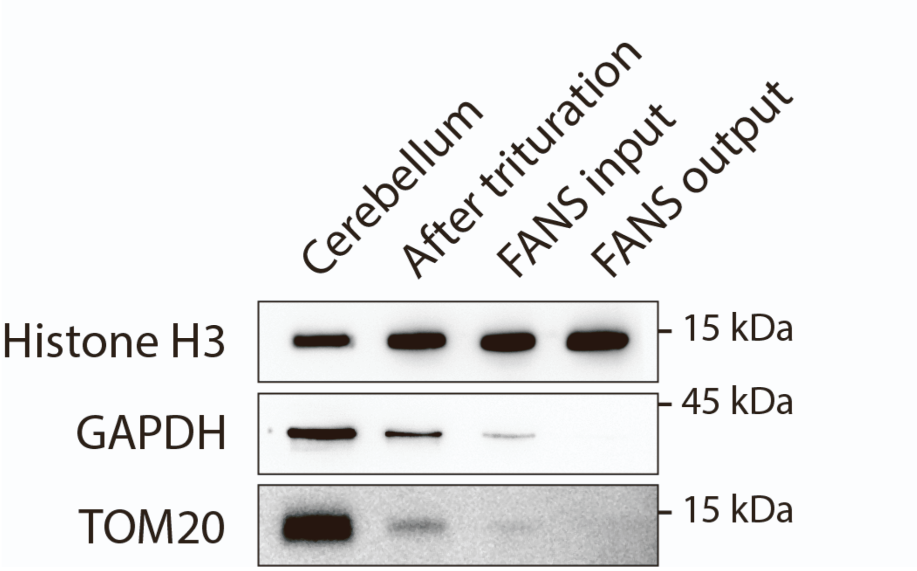

Supplementary Figure 2. The quality of nuclear fractionation assessed through Western blot analysis, related to the STAR Method - Western blotting.
Samples were obtained at various stages of the isolation protocol, including unlysed tissue, the fraction after trituration, the input fraction for FANS, and the output fraction from FANS. Equal protein amounts were loaded onto a gel for each fraction. The observations revealed a gradual decrease in cytosolic markers (GAPDH) and mitochondrial markers (TOM20) until they were no longer detectable in the FANS output fraction. Simultaneously, there was a gradual increase in the nuclear marker Histone H3, indicating the efficient removal of cytosolic components.

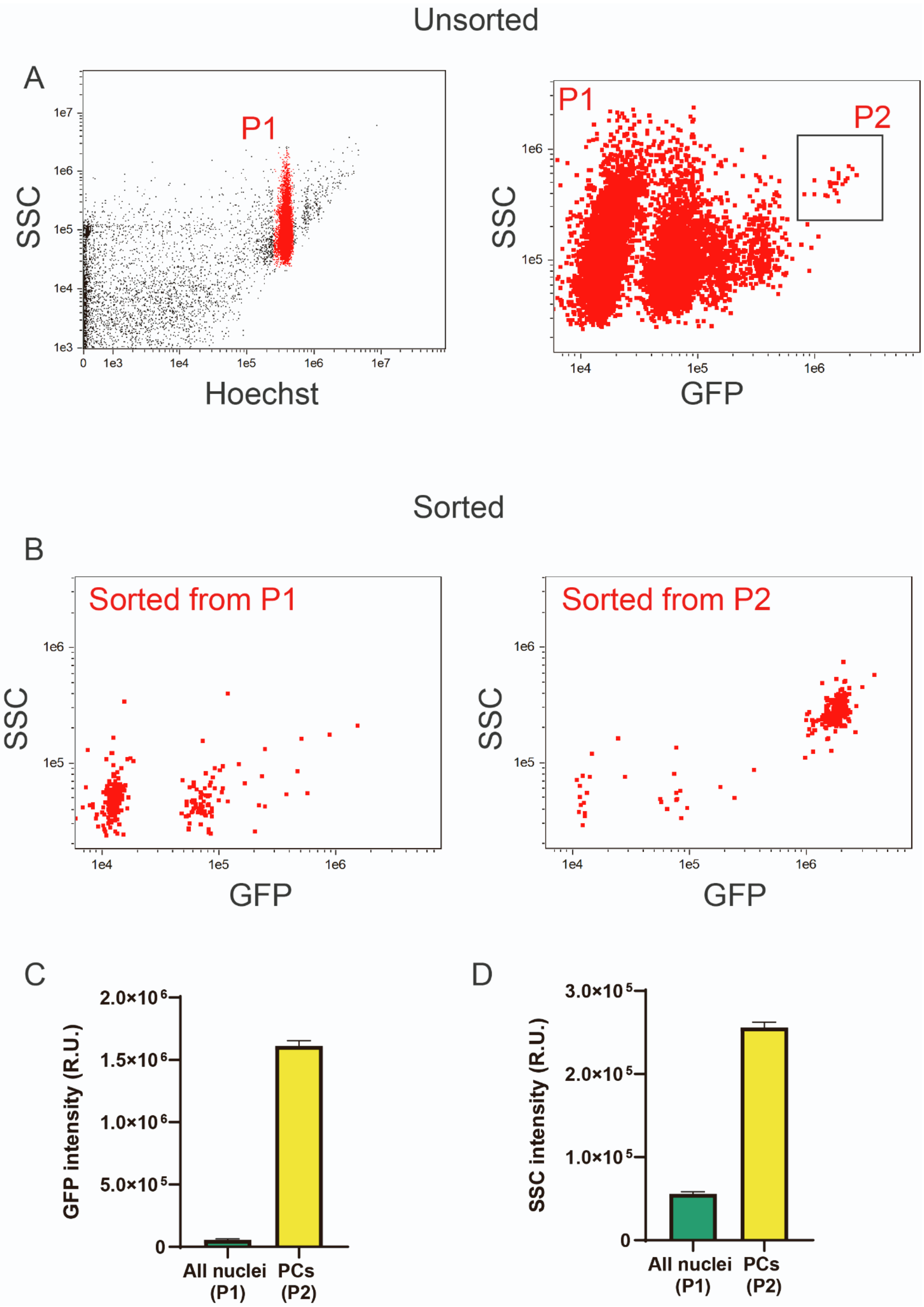

Supplementary Figure 3. ImageStream cytometric analysis of nuclei isolated from Sun1/sfGFP-positive and Pcp2-Cre-positive cerebella, related to Figure 2.

A) Both unsorted nuclei and B) nuclei previously sorted on the BD FACSAria Fusion flow cytometer were included in this analysis. C) The bar graphs illustrate variations in GFP intensity and D) side scatter (SSC) intensity, as computed by the IDEAS software, within the P1 and P2 populations indicated on the plots above.

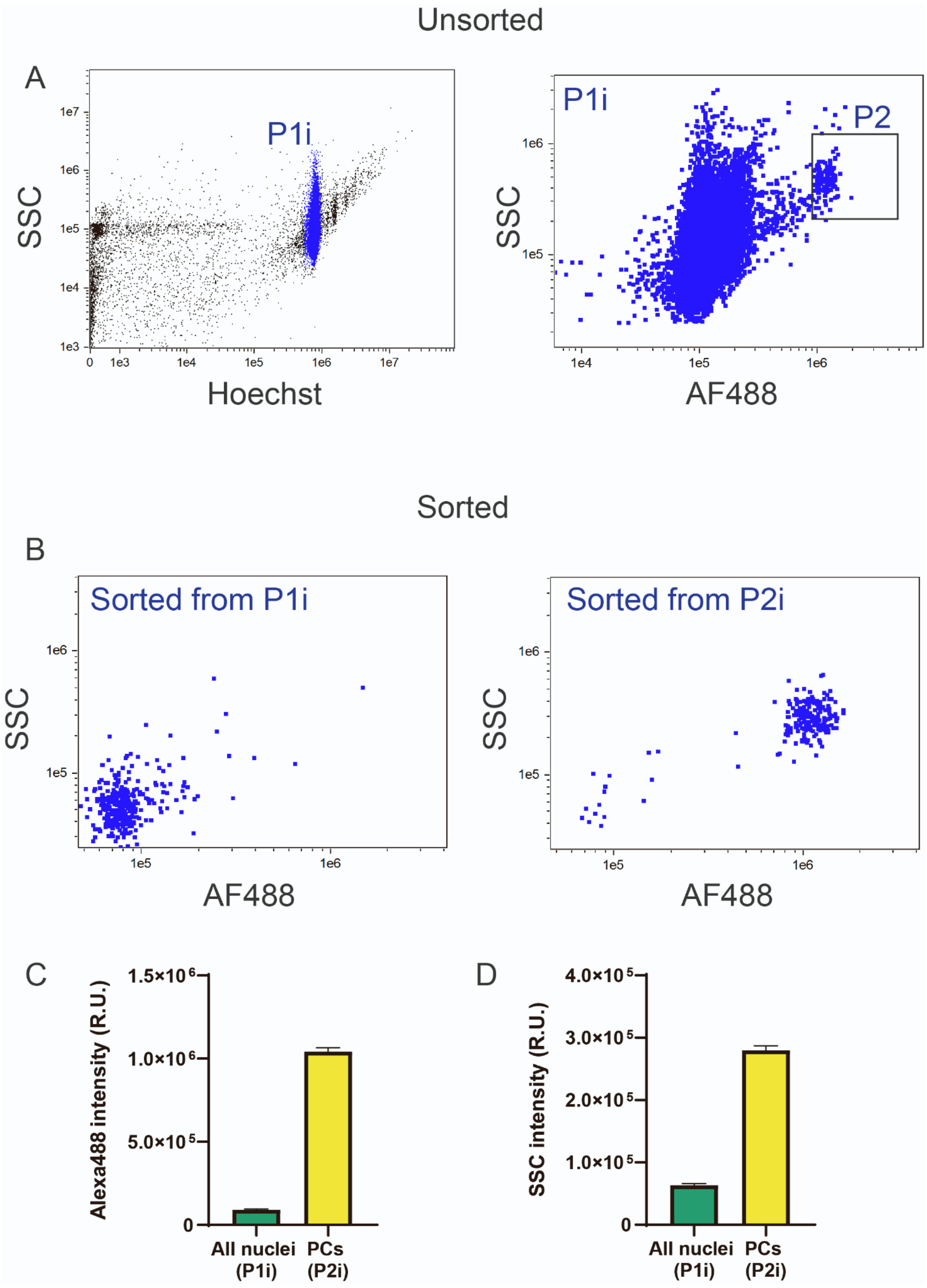

Supplementary Figure 4. ImageStream cytometric analysis performed on nuclei immunostained against RanBP2, which were isolated from wild-type cerebella, related to Figure 4.

A) Both unsorted nuclei and B) nuclei previously sorted on the BD FACSAria Fusion flow cytometer were included in this analysis. C) The bar graphs illustrate variations in AlexaFluor488 intensity and D) side scatter (SSC) intensity, as computed by the IDEAS software, within the P1i and P2i populations indicated on the plots above.

Supplementary Table 1: Contribution of PC Nuclei to Overall Singlet Nuclei (%), related to Figures 1 and 3.

% of PC in singlets	Sun1_GFP	RanBP2
exp_1	0.5	0.8
exp_2	0.4	1
exp_3	0.5	0.6
exp_4	0.4	0.9
exp_5	0.2	0.7
exp_6	0.3	0.6
exp_7	0.5	0.6
exp_8	0.3	0.5
exp_9	0.4	0.7
exp_10	0.3	0.3
exp_11	0.5	0.3
exp_12	0.3	0.7
exp_13	0.4	0.8
exp_14	0.5	0.5
exp_15	0.5	0.7
Average	0.40	0.65
