# Supplementary material for: Antibody-assisted selective isolation of Purkinje cell nuclei from mouse cerebellar tissue
Source: Cell Rep Methods. 2024 Jul 8;4(7):100816. doi: 10.1016/j.crmeth.2024.100816 (PMC11294835; doi:10.1016/j.crmeth.2024.100816)
Supplement: Document S2. Article plus supplemental information [file mmc2.pdf]

Cell Reports Methods

Report

Antibody-assisted selective isolation of Purkinje cell nuclei from mouse cerebellar tissue

Graphical abstract

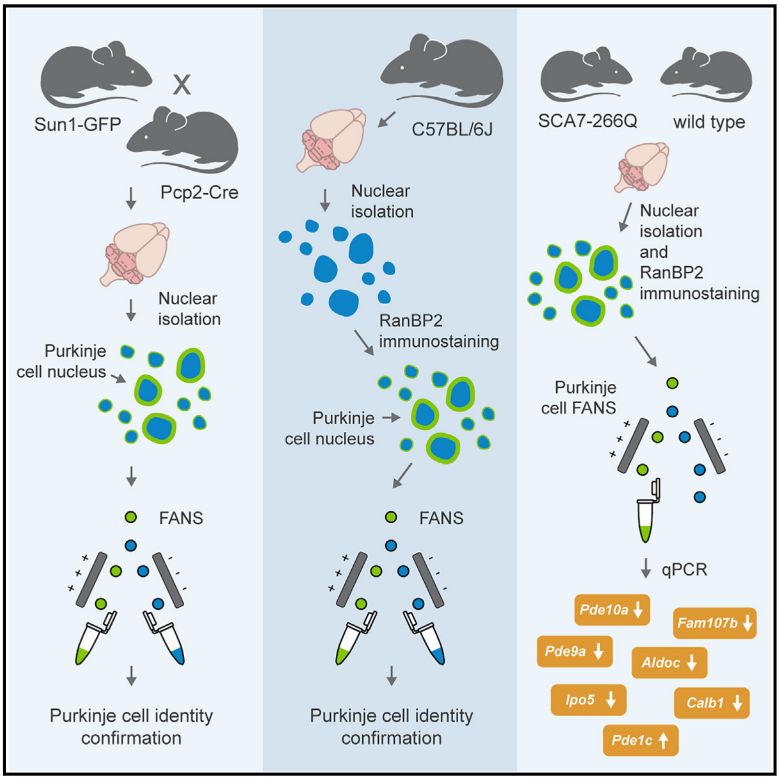

Highlights

- We present a quick and inexpensive method to selectively isolate Purkinje cell nuclei
- Our protocol combines fluorescence labeling of nuclear envelopes with FANS
- We provide thorough confirmation that the isolated nuclei have Purkinje cell identity
- The protocol aids in studying neurodegeneration mechanisms in spinocerebellar ataxias

Authors

Luke C. Bartelt, Mouad Fakhri, Grazyna Adamek, ..., Craig B. Lowe, Albert R. La Spada, Pawel M. Switonski

Correspondence

alaspada@uci.edu (A.R.L.S.), pswiton@ibch.poznan.pl (P.M.S.)

In brief

Bartelt et al. introduce a protocol for fluorescently labeling nuclear envelopes, paired with cell sorting to purify Purkinje cell nuclei. By providing access to the nuclear fraction of these rare neurons, the method offers a valuable platform for studying Purkinje cell biology and pathology, including the mechanism behind selective neurodegeneration.

Bartelt et al., 2024, Cell Reports Methods *4*, 100816
July 15, 2024 © 2024 The Author(s). Published by Elsevier Inc.
https://doi.org/10.1016/j.crmeth.2024.100816

CellPress

Cell Reports Methods

CellPress
OPEN ACCESS

Report

Antibody-assisted selective isolation of Purkinje cell nuclei from mouse cerebellar tissue

Luke C. Bartelt,[1,2,3,9] Mouad Fakhri,[4,9] Grazyna Adamek,[4] Magdalena Trybus,[5] Anna Samelak-Czajka,[5] Paulina Jackowiak,[5] Agnieszka Fiszer,[6] Craig B. Lowe,[3] Albert R. La Spada,[2,7,8,*] and Pawel M. Switonski[4,10,*]
[1]University Program in Genetics & Genomics, Duke University Medical Center, Durham, NC 27710, USA
[2]Departments of Pathology & Laboratory Medicine, Neurology, Biological Chemistry, and Neurobiology & Behavior, University of California, Irvine, Irvine, CA 92697, USA
[3]Department of Molecular Genetics and Microbiology, Duke University Medical Center, Durham, NC 27710, USA
[4]Department of Neuronal Cell Biology, Institute of Bioorganic Chemistry, Polish Academy of Sciences, 61-704 Poznan, Poland
[5]Laboratory of Single Cell Analyses, Institute of Bioorganic Chemistry, Polish Academy of Sciences, Noskowskiego 12/14, 61-704 Poznan, Poland
[6]Department of Medical Biotechnology, Institute of Bioorganic Chemistry, Polish Academy of Sciences, 61-704 Poznan, Poland
[7]Department of Neurology, Duke University School of Medicine, Durham, NC 27710, USA
[8]UCI Center for Neurotherapeutics, University of California, Irvine, Irvine, CA 92697, USA
[9]These authors contributed equally
[10]Lead contact
*Correspondence: alaspada@uci.edu (A.R.L.S.), pswiton@ibch.poznan.pl (P.M.S.)
https://doi.org/10.1016/j.crmeth.2024.100816

MOTIVATION Cerebellar Purkinje cells (PCs) play a central role in controlling voluntary movements, coordination, and motor learning. PC degeneration and death are hallmarks of many human neurological disorders, including multiple forms of spinocerebellar ataxia. Unfortunately, PCs comprise a relatively small population of cells in the cerebellum, constituting less than 1% of the total cell count. This makes studying PC-related pathology using omics approaches exceptionally challenging. Here, we introduce a protocol that addresses the need for obtaining pure fractions of PC nuclei for subsequent cell-specific analysis.

SUMMARY

We developed a method that utilizes fluorescent labeling of nuclear envelopes alongside cytometry sorting for the selective isolation of Purkinje cell (PC) nuclei. Beginning with SUN1 reporter mice, we GFP-tagged envelopes to confirm that PC nuclei could be accurately separated from other cell types. We then developed an antibody-based protocol to make PC nuclear isolation more robust and adaptable to cerebellar tissues of any genotypic background. Immunofluorescent labeling of the nuclear membrane protein RanBP2 enabled the isolation of PC nuclei from C57BL/6 cerebellum. By analyzing the expression of PC markers, nuclear size, and nucleoli number, we confirmed that our method delivers a pure fraction of PC nuclei. To demonstrate its applicability, we isolated PC nuclei from spinocerebellar ataxia type 7 (SCA7) mice and identified transcriptional changes in known and new disease-associated genes. Access to pure PC nuclei offers insights into PC biology and pathology, including the nature of selective neuronal vulnerability.

INTRODUCTION

Cerebellar Purkinje cells (PCs) are GABAergic autonomous neurons that exhibit high rates of spiking even in the absence of synaptic input. PCs control voluntary movements by integrating enormous amounts of synaptic input from various brainstem and cerebellar neurons.[1] Being the sole output of cerebellar cortex, PCs play a critical role in motor coordination and learning.[2] PC dysfunction and death are frequently observed in patients with ataxia and in animal models with ataxic symptoms. A list of "purkinjopathies" includes, but is not limited to, multiple forms of spinocerebellar ataxias (SCAs), Friedreich's ataxia, episodic ataxia type 2, Niemann-Pick disease type C, and PC degeneration.[3] Although the PC selective vulnerability that characterizes these disorders has been the subject of extensive research, molecular mechanisms responsible for selective degeneration still await a satisfactory explanation.

This is an open access article under the CC BY license (http://creativecommons.org/licenses/by/4.0/).

PCs are notoriously difficult to isolate due to their complex morphology and low abundance in the cerebellum (less than 1% of cells). Laser capture microdissection proved to be a valuable, although costly and laborious, technique to isolate PCs for downstream analysis.[4] Some groups have attempted to isolate PCs using fluorescence-activated cell sorting (FACS) and GFP reporter mice.[5,6] Even mild enzymatic digestion, however, inevitably compromises the integrity of PC long axons and intricately branched dendritic trees, severely limiting the utility of FACS. Additionally, advancement of cell-type-specific ribosome tagging protocols helped survey the molecular landscape of PC subcellular domains.[7] Although very potent and high throughput, ribosome tagging preserves only transcriptional information of the molecular landscape of the cell and requires transgene delivery by adeno-associated virus. Because PCs are difficult to isolate from complex brain tissue, bulk tissue profiling is used to generate the majority of molecular data aimed at understanding PC vulnerability. Bulk approaches average out the signal from affected cells, undermining our understanding of the cell-type-specific pathogenic mechanisms responsible for selective PC neurodegeneration.

To bypass the limitation of destructive PC purification while still retaining epigenetic, transcriptomic, genetic, and nucleoproteomic profiles of isolated cells, we chose to establish a method for PC-specific nuclear fractionation. We begin by confirming the effectiveness of the genetic labeling of nuclear envelopes, followed by nuclear isolation and fluorescence-activated nucleus sorting (FANS), in generating a highly enriched population of PC nuclei. Building upon this foundation, we introduce an antibody-based FANS purification method that provides a unique solution for the efficient and selective isolation of PC nuclei.

RESULTS

FANS-based isolation of PCP2-Cre x SUN1-GFP mouse PC nuclei

Given the significant heterogeneity of the cellular and nuclear morphology between cerebellar cell types, we sought to separate PC nuclei from other cell nuclei with FANS. We crossed CAG-Sun1/superfolder GFP (sfGFP) reporter mice, developed by Mo et al. for the isolation of nuclei tagged in specific cell types application, with mice expressing Cre recombinase driven by the PC-specific Pcp2 promoter to drive PC-specific expression of GFP-tagged nuclear envelope protein SUN1.[8] To confirm successful envelope tagging, we cryosectioned and immunostained cerebella from adult 8.5-week-old mice with an anti-myc antibody recognizing the Sun1-GFP transgene. As expected, control littermates with Sun1/sfGFP^{+}, Pcp2-Cre^{-}genotypes showed no immunofluorescent signal originating from nuclear envelopes. In contrast, Sun1/sfGFP^{+}, Pcp2-Cre^{+} mice displayed strong nuclear membrane staining that was dominant but not limited to PCs (Figure 1A). To separate PC nuclei from the rest of GFP^{+} cerebellar cell nuclei, we isolated nuclear fractions from Sun1/sfGFP^{+}, Pcp2-Cre^{+} mice using a modified mechanical trituration protocol described by Kozareva and colleagues.[9] Next, we counterstained isolated nuclei with Hoechst and subjected them to flow cytometry analysis. To exclude cell debris, nuclear aggregates, and other unwanted particles from the subsequent analysis, we gated single nuclei according to their Hoechst intensity, shown as population P1 (Figures 1B and S1). Next, we analyzed P1 nuclei for their GFP intensity and side scatter (SSC) profiles. The nuclei with the strongest GFP signal also separated out due to their increased side scattering, forming a distinct cluster in the SSC/GFP dot-plot space (Figure 1C). This population, labeled P2, constituted 0.4% of the overall nuclei (Table S1). To evaluate the purity of our sorting strategy, we sorted the P2 population, performed post-sort analysis, and found 89.2% of P2 nuclei among all nuclei in sorted samples (Figure 1D). Finally, to confirm the quality of our nuclear fractionation, we performed western blot analysis. Samples were collected at different stages of the nuclear fractionation process, such as unlysed tissue, triturated fraction, FANS input, and FANS output. We observed a progressive decline in cytosolic (glyceraldehyde 3-phosphate dehydrogenase) and mitochondrial (TOM20) markers until they were undetectable in the FANS output. Concurrently, there was a gradual rise in the nuclear marker histone H3, suggesting effective removal of cytosolic components (Figure S2).

Nuclei isolated from PCP2-Cre x SUN1-GFP mouse cerebella display PC identity

To confirm PC identity of the P2 population, we sorted 4,000 P1 and P2 nuclei and performed qRT-PCR to test for the expression of PC marker genes. We observed a dramatic increase in the expression of *Calb1* (144.9-fold), *Ppp1R17* (130.4-fold), *Pcp2* (95.2-fold), and *Ryr1* (271.8-fold) in the sorted P2 nuclei (Figure 2A). In contrast, the granule cell neuron-specific marker *Gabra-6* was significantly depleted in the P2 fraction (Figure 2B). Next, we inspected P1 and P2 populations on the ImageStream flow imager to assess nuclear morphology. High content image analysis uncovered striking differences in size, GFP fluorescent intensity, and SSC magnitude between the two populations (Figure 2C). The median P2 nucleus size was 131.8 μm^2, compared to 69.4 μm^2 for P1 (Figure 2D). We also detected significantly higher average intensity in GFP (29-fold change) and SSC (4.6-fold change) in P2 nuclei (Figure S3). Previous studies have found that a characteristic feature of developed mouse PCs is a single, centrally located nucleolus.[10] To determine the number of nucleoli in P1 and P2 populations, we stained sorted nuclei with the Nucleolus Bright Red (NBR) dye. We calculated that an overwhelming 90.9% of P2 nuclei, but only 49.7% of P1 nuclei with NBR signal, contained a single nucleolus (Figure 2E). These findings confirm the PC identity of the P2 population and demonstrate that our nuclear isolation approach provides a reliable and effective way to isolate a pure fraction of PC nuclei.

RanBP2 immunolabeling, isolation, and identity confirmation of wild-type (WT) mouse PC nuclei

To improve the method and adapt it to various tissue sources, we then developed an immunofluorescence labeling protocol. We decided to target RanBP2, a filament protein found on the cytoplasmic side of the nuclear pore complex that displays elevated expression in mouse and human PCs.[11,12] To immunolabel isolated nuclei, we incubated 8.5-week-old WT mouse cerebellar nuclei with an anti-RanBP2-AlexaFluor488 antibody. When we examined single immunotagged nuclei on the flow cytometer, we discovered a subpopulation with high Alexa Fluor 488 and

CellPress
OPEN ACCESS

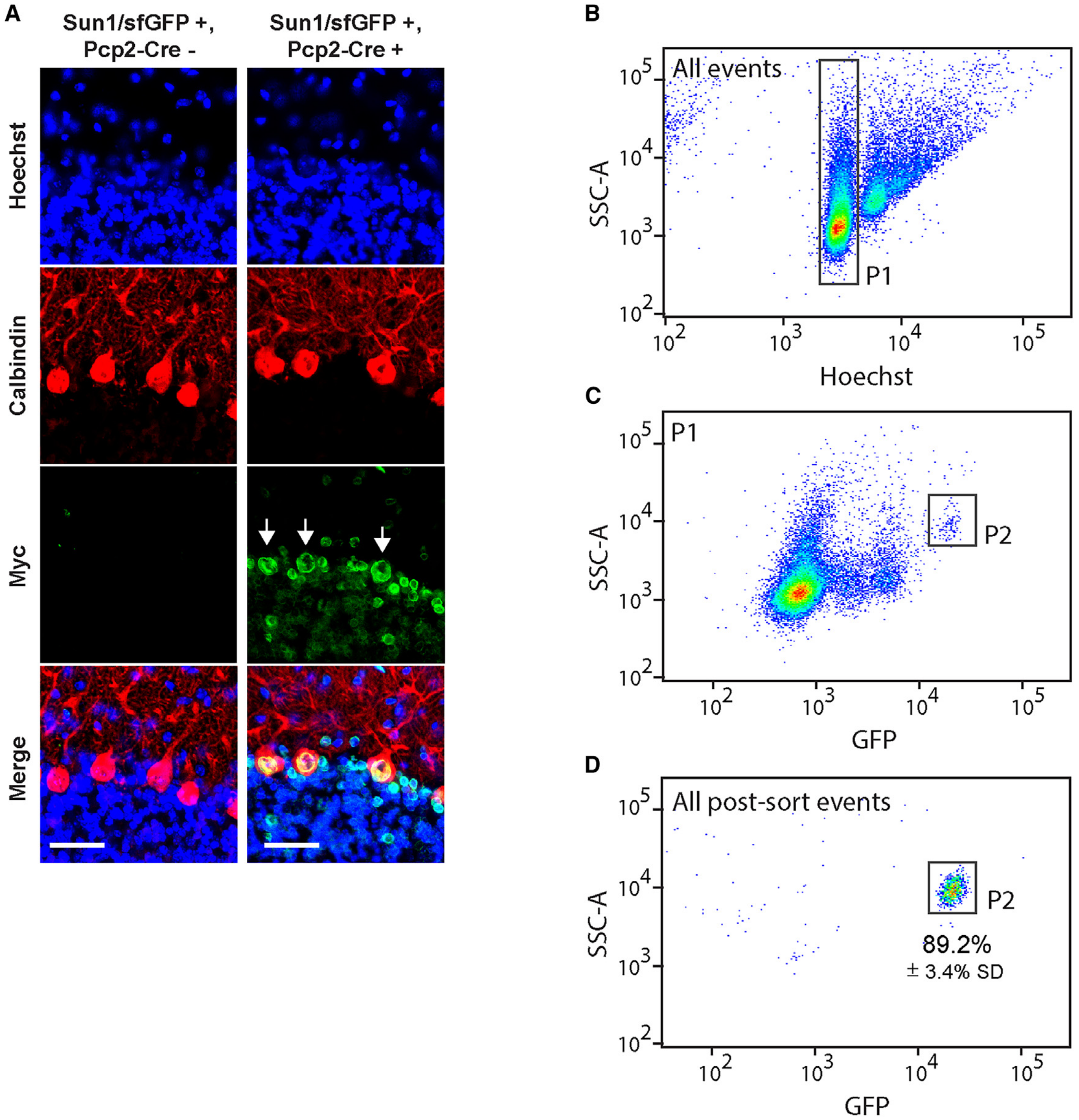

Figure 1. SUN1-GFP genetic tagging of nuclear envelopes, followed by flow cytometry analysis, reveals a distinctive cluster of PC nuclei

(A) We cryosectioned cerebella from Sun1/sfGFP$^+$, Pcp2-Cre$^-$ and Sun1/sfGFP$^+$, Pcp2-Cre$^+$ mice and immunostained them for the Myc tag (green), which is fused to the GFP protein, calbindin (red), and Hoechst (blue). The arrows point to the PC nuclei. Scale bar: 50 μm.

(B) We loaded nuclei isolated from Sun1/sfGFP$^+$, Pcp2-Cre$^+$ cerebella into a cell sorter, and used SSC-A vs. Hoechst-A to identify the population of single nuclei (called P1).

(C) We examined P1 nuclei for SSC-A and GFP-A and discovered several subpopulations, one of which was distinguished by strong SSC and GFP intensity (called P2).

(D) We performed post sort analysis to ensure P2 sorting purity and found 89.2% of P2 nuclei among all nuclei in sorted samples; $n = 3$ independent purifications. Hoechst-A, Hoechst channel peak area; P1 (population 1), all singlet nuclei; P2 (population 2), PC nuclei.

SSC intensities (Figures 3A and 3B). That nuclear population (called P2i) was strikingly similar to the P2 population obtained via Sun1/sfGFP genetic tagging. To determine the purity of the sorted P2i nuclei, we performed post-sort analysis on three independent preparations and discovered 94.2% pure P2i nuclei with limited contamination (Figure 3C). To verify the PC identity of P2i nuclei, we determined the expression of PC markers in 4,000 P1i and P2i nuclei. As in the Sun1/sfGFP experiment, *Calb1*, *Pcp2*, *Ryr1*, and *Ppp1R17* expression levels in P2i were markedly elevated. We observed an 85.6-fold increase in *Calb1*, a 114.8-fold increase in *Ppp1R17*, a 60.6-fold increase in *Pcp2*, and a 394.1-fold increase in *Ryr1* expression (Figure 4A). In contrast, the P2i fraction was depleted of the granule cell neuron marker *Gabra-6* (Figure 4B). Next, we subjected sorted P1i and P2i populations to ImageStream high content image analysis and noted increased SSC (4.4-fold change) and Alexa

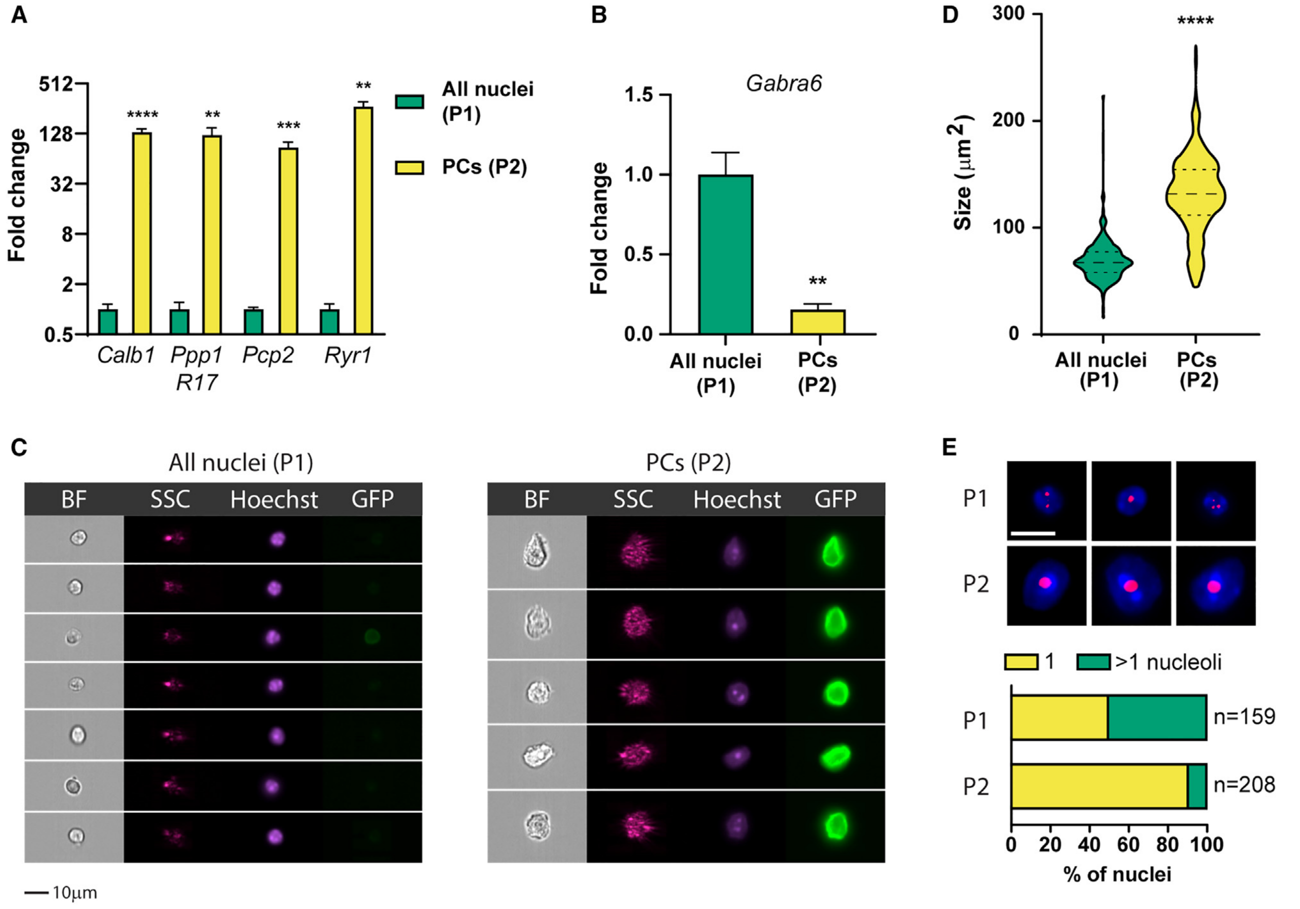

Figure 2. Morphological and molecular analysis of the P2 subset from Sun1/sfGFP cerebella confirms its PC identity

(A) We used qRT-PCR to compare the expression of the most specific PC marker genes in 4,000 P1 and P2 nuclei sorted from Sun1/sfGFP$^+$, Pcp2-Cre$^+$ cerebella; n = 3 for each group.

(B) qRT-PCR quantification of *Gabra6*, a marker of cerebellar granule neurons; n = 3 for each group.

(C) We imaged P1 and P2 populations by reloading previously sorted P1 and P2 nuclei into the ImageStream cytometer. Representative examples are shown; scale bar: 10 μm.

(D) Nuclear size distribution in P1 and P2 populations represented as a Hoechst footprint in the ImageStream analysis; n = 227 and 237 for P1 and P2, respectively.

(E) We stained nuclei isolated from Sun1/sfGFP$^+$, Pcp2-Cre$^+$ cerebella with nucleolar dye NBR, counterstained them with Hoechst, and visualized both P1 and P2 populations with the ImageStream cytometer. Representative examples are shown. Scale bar: 10 μm. Quantification of the number of nucleoli per cell is shown below. Data are represented as mean ± SEM.

Fluor 488 (11.5-fold change) intensities of P2i nuclei (Figures 4C and S4). We also recorded a median size of 59.1 and 135.2 μm^2 for P1i and P2i nuclei, respectively, which is remarkably consistent with the P1 and P2 populations collected in the genetic tagging experiments (Figure 4D). To count the number of nucleoli, we stained both P1i and P2i populations with NBR. We found that 91.3% of NBR$^+$ P2i nuclei had a single nucleolus, in contrast to 50.2% of P1i (Figure 4E). Our findings show that the P2 and P2i populations are equivalent and represent a pure fraction of PC nuclei.

PC nuclear isolation provides a platform for disease-associated gene expression measurements in SCA7 mice

To further validate the specificity of our antibody-assisted isolation method, we sought to verify transcriptional dysregulation in PC nuclei isolated from SCA7 mice. We used SCA7-266Q mice, an established knockin genetic model of SCA7 that faithfully reproduces rapidly progressive neurodegeneration, with selective vulnerability of cerebellar PCs.[13] Our objective was to verify the expression levels of several genes, previously established to be downregulated in other SCA7 mouse models,[14,15] as well as in a single-cell RNA sequencing (RNA-seq) experiment previously conducted by our team.[16] Using the RanBP2 immunolabeling approach, we isolated and sorted 4,000 PC nuclei from symptomatic 10-week-old SCA7-266Q mice and WT littermates. To assess differences in gene expression between SCA7 and WT PCs, we employed qRT-PCR to measure the expression levels of *Aldoc*, *Fam107b*, *Ipo5*, and *Calb1* alongside several phosphodiesterases (PDEs), namely *Pde1c*, *Pde4d*, *Pde9a*, and *Pde10a*. PDEs were chosen for screening due to their pivotal role in the cyclic nucleotide signaling pathway, which has been observed to deteriorate in

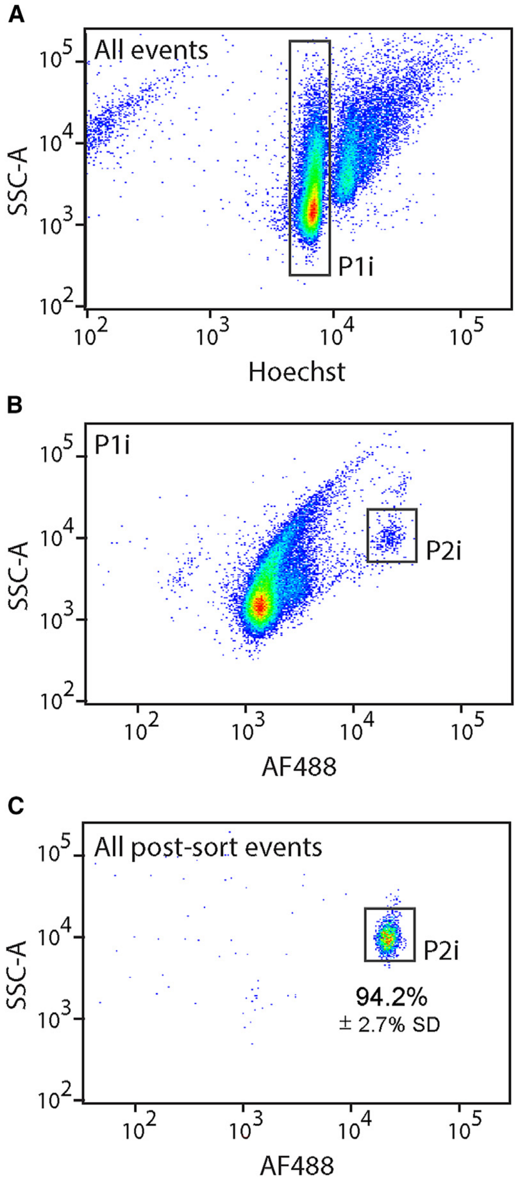

Figure 3. Flow cytometry analysis of RanBP2-stained cerebellar nuclei reveals a distinct subpopulation of PC nuclei

(A) We analyzed nuclear extracts from mouse cerebellum with a cell sorter, and identified single nuclei (dubbed P1i) using SSC-A vs. Hoechst-A.

(B) Examining the P1i singlets for SSC-A and AlexaFluor488-A revealed distinct subpopulations, including one prominent P2i cluster with strong side scattering and Alexa Fluor 488 intensity.

(C) We reexamined sorted P2i nuclei to ensure purity. Three independent preparations revealed that 94.2% of sorted nuclei fell back into the P2i gate. AlexaFluor488-A, Alexa Fluor 488 channel peak area; Hoechst-A, Hoechst channel peak area; P1i (population 1, immunostained), all singlet nuclei; P2 (population 2, immunostained), PC nuclei.

aging brains[17,18] and in Alzheimer disease (AD),[19,20] Parkinson disease (PD),[21] and Huntington disease (HD).[22,23]

In our screen of SCA7 PC nuclei, we observed markedly decreased expression of *Aldoc*, *Fam107b*, *Ipo5*, *Calb1*, *Pde9a*, and *Pde10a*, as well as increased expression of *Pde1c* (Figure 5A). These findings confirm previous observations of transcriptional dysregulation of these genes in SCA7 and prompt further investigation into whether PC-specific transcriptional dysregulation may contribute to the selective neuronal vulnerability observed in SCA7 and related neurodegenerative disorders.

DISCUSSION

Despite decades of research indicating that selective vulnerability of PCs is a common theme in many SCAs and other cerebellar conditions, technical limitations have prevented researchers from easily isolating PCs for detailed study. To better understand the extrinsic and intrinsic factors that govern PC susceptibility to death, detailed molecular characterization of PC physiology and pathophysiology is required.[24] Here, we present methods for genetic and immunofluorescence nuclear labeling, followed by FANS isolation to produce a large number of pure PC nuclei. Because nuclei retain genetic, epigenetic, and transcriptomic information of cells, our method can be applied to a variety of analyses aimed at better understanding PC-dependent pathological mechanisms in SCAs and other diseases. Furthermore, our protocol will be equally applicable to studies of the basic science underlying cerebellar development and function. Moreover, we demonstrated the utility of our PC nuclei isolation protocol by documenting transcriptional alterations in SCA7 Purkinje cells. This included changes in genes previously linked to the disease (*Aldoc*, *Fam107b*, *Ipo5*, *Calb1*), as well as dysregulation of genes associated with the cyclic nucleotide signaling pathway (*Pde1c*, *Pde9a*, *Pde10a*).

To develop an antibody-based method for labeling and isolating PC nuclei, we began with a previously established genetic approach. By crossing mice expressing conditionally regulated Sun1-GFP with mice expressing Cre recombinase driven by the PC-specific Pcp2 promoter, we established bi-genic animals with genetically tagged nuclear envelopes. The general Sun1-GFP tagging approach was developed by Mo et al., and has since been used to isolate rare cellular populations, including excitatory pyramidal neurons, parvalbumin and vasoactive intestinal peptide-expressing interneurons,[8] sensory ganglia neurons,[25,26] oligodendrocyte progenitor cells,[27] nucleus accumbens interneurons,[28] septal neurons,[29] forebrain principal neurons,[30] and glutamatergic projection neurons.[31]

During the preparation of this manuscript, Chen and colleagues published their work on PC diversification during the learning process, and employed the same Sun1-GFP x Pcp2-Cre labeling method to isolate PC nuclei from mouse cerebella.[32] In contrast to their report, we observed Sun1-GFP expression not only in PCs but also in other cell types. These differences may be explained by the different Pcp2-Cre driver lines used in each study.

In our research, we utilized the Pcp2-Cre Mpin line, initially developed by Barski et al..[33] This line was created by inserting the Cre cDNA into the Pcp2 minigene, which was subsequently introduced into embryonic stem cells via electroporation. Unlike methods relying on homologous recombination-mediated targeted insertion, this approach resulted in the integration of a few transgene copies. Analysis of the specificity of Cre

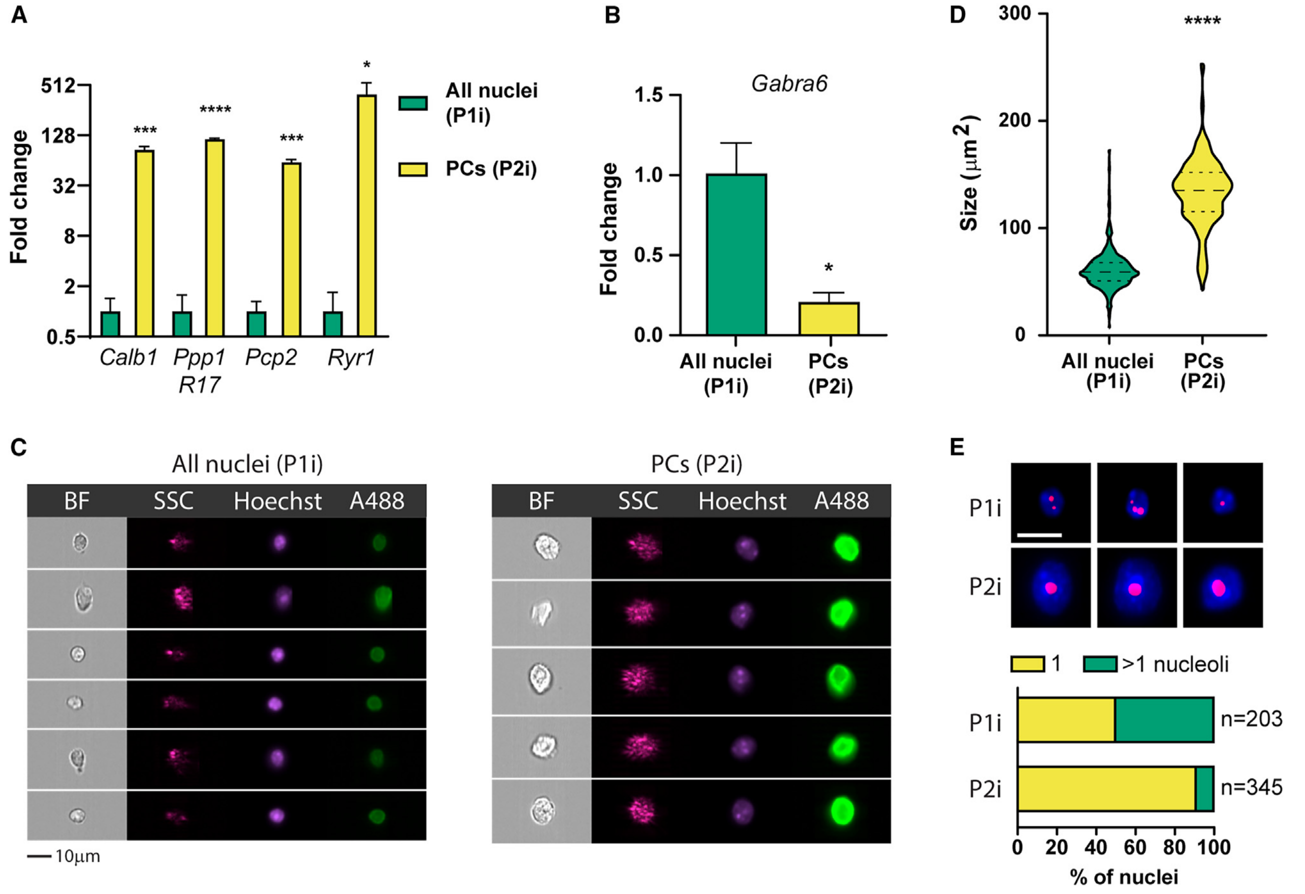

Figure 4. Morphological and molecular characterisation of RanBP2-stained P2i subset confirms its PC identity

(A) We sorted 4,000 P1i and P2i nuclei and compared the expression of the most specific PC marker genes with qRT-PCR; $n = 3$ for each group.

(B) qRT-PCR quantification of *Gabra6*, a marker of cerebellar granule neurons; $n = 3$ for each group.

(C) We visualized P1i and P2i previously sorted nuclei by reloading them into the ImageStream cytometer. Representative examples are shown. Scale bar: 10 μm.

(D) Nuclear size distribution in P1i and P2i populations represented as a Hoechst footprint in the ImageStream analysis. n = 320 and 197 for P1i and P2i, respectively.

(E) We stained isolated nuclei with the nucleolar dye NBR, counterstained them with Hoechst, and visualized both P1i and P2i populations with the ImageStream cytometer. Scale bar: 10 μm. Representative examples are shown. Quantification of the number of nucleoli per cell is shown below. Data are represented as mean ± SEM.

expression revealed robust transgene expression in PCs, with minimal expression observed in retinal bipolar neurons. However, a small proportion (<5%) of randomly distributed cells with Cre expression were documented in the cerebellar cortex, as well as in other brain regions and non-neural tissues.[33,34]

An alternative line, the Pcp2-Cre Jdhu line developed by Huang's group, was designed to carry the entire Pcp2 gene with the Cre cDNA inserted into a large bacterial artificial chromosome.[35] This design ensured that the Jdhu line retained all potentially crucial regulatory elements responsible for the PC-specific Pcp2-Cre expression, which the Mpin line lacks. This distinction likely accounts for the greater expression specificity observed with the Jdhu line.[34,35] Nevertheless, the considerable level of non-specific Cre expression detected in our mice implies that factors beyond Mpin-related background also played a role in the reduced specificity. It is frequently observed that unaccounted, transient Cre expression in the germ line or during early development can trigger recombination events, reducing specificity for the target population.[36] Because of the wide range of Sun1-GFP expression in our Sun1/sfGFP^{+}, Pcp2-Cre^{+} mice, we evaluated isolated nuclei, not only by GFP intensity but also by SSC magnitude, which turned out to be a useful indicator for cerebellar nuclear morphology. Following a series of morphological and molecular tests conducted on the PC nuclei population, we have successfully established a set of reference criteria for PC identity validation. Moreover, we realized that if PC nuclei can be distinguished in the GFP/SSC dot-plot space despite the widespread expression of Sun1-GFP in cerebellar cells, genetic nucleus tagging could be replaced with simple immunostaining.

We chose RAN binding protein 2 (RanBP2, also known as NUP358) as a target for nuclear envelope antibody-based labeling. RanBP2 is a large protein that is a component of cytoplasmic fibrils and resides on the cytoplasmic side of the nuclear pore complex.[37] Importantly, both mouse and human brain atlases

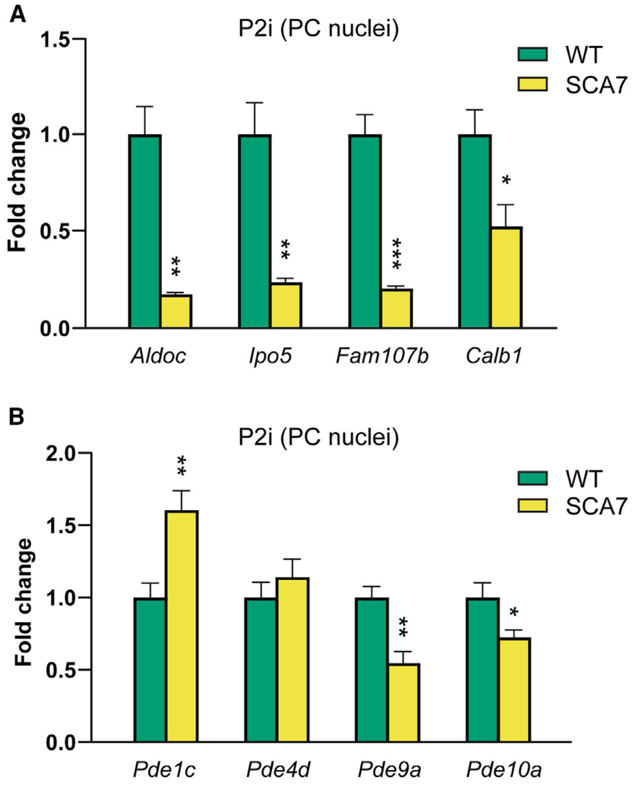

Figure 5. qPCR quantification in SCA7 PC nuclei unveils transcriptional changes in both known and new disease-associated genes

(A) qPCR quantification of selected disease-linked genes in PC nuclei isolated from SCA7 and WT mouse cerebella. $n = 4$ cerebella for each group; 4,000 nuclei were sorted per animal.

(B) qPCR quantification of *Pde1c*, *Pde4d*, *Pde9a*, and *Pde10a* PC nuclei isolated from SCA7 and WT mouse cerebella. $n = 7$ cerebella for each group; 4,000 nuclei were sorted per animal. Data are represented as mean ± SEM.

indicate increased RanBP2 expression in PCs.[11,12] As expected, RanBP2 staining of mouse cerebellar nuclei produced a distinct population of PC nuclei that has important implications for future PC research. PC nuclei can be easily isolated using a quick, simple, and inexpensive protocol that does not require a complicated process of transgenic mouse breeding. Interestingly, assuming that the intensity of the FANS signal generated by an evenly labeled nuclear envelope is primarily driven by nuclear surface area, immunotargeting other nuclear pore complex components or LINC (linker of nucleoskeleton and cytoskeleton) members[38] should also result in successful PC nucleus segregation. Furthermore, since proper FACS gating allows for the isolation of PC nuclei even in high backgrounds of off-target cell types, it paves the way for exploring this method in virus-based labeling approaches.

To confirm the PC identity of isolated nuclei, we performed a battery of molecular and morphological evaluations, mirroring those carried out for genetically tagged PC nuclei. ImageStream analysis enabled us to visually inspect PC nuclei and quantify their size. PC nuclei were found to be significantly larger in size than other cerebellar cell nuclei, which is consistent with previous findings in cerebellar tissue.[39] Similarly, we used nucleoli number as a key feature to distinguish PCs from other cerebellar cell types. Previous studies have found that the average number of nucleoli per nucleus in mouse PCs decreases steadily during postnatal development, eventually reaching one in adult cells.[10] We discovered that >90% of PC nuclei had only one nucleolus, in contrast to other nuclei isolated from cerebellar tissue. Finally, we reinforced our PC evaluation by looking at the enrichment of PC-specific marker expression in PC nuclei. *Calb1*, *Pcp2*, *Ryr1*, and *Ppp1r17*—four genes with significantly increased expression in our PC nuclei—are highly specific PC markers, as demonstrated by single-cell sequencing data.[9] An important aspect of our analysis, which supports the PC identity of isolated nuclear populations, is that both genetic and immune-based methods of nuclear envelope tagging produced remarkably similar morphological and transcriptional evaluation outcomes.

Transcriptional dysregulation is a recurring theme in many neurodegenerative diseases.[40–44] This is particularly evident in SCA7, where the causative protein ataxin-7 is a cofactor in the multiprotein high-molecular-weight SPT3-TAF9-GCN5 acetyltransferase chromatin remodeling complex.[45,46] Our own chromatin immunoprecipitation sequencing H3K9 acetylation and H2BK120 ubiquitination profiling data from mouse retinal and cerebellar tissues indicate widespread epigenetic changes in SCA7 animals.[47,48] To demonstrate the applicability of our PC nuclei isolation method, we purified the PC nuclei from SCA7-266Q mice and assessed them for dysregulated expression of known SCA7-linked genes. These genes, previously established to be transcriptionally dysregulated in other studies, included aldolase C (*Aldoc*) and importin-5 (*Ipo5*), whose expression was significantly diminished in both bulk RNA-seq of SCA7-140Q knockin mice and in our single-cell analysis.[15,16] We also investigated *Fam107b* and *Calb1*, which exhibited downregulation across three independent studies: in SCA7-140Q mice, in single-cell datasets, and in bulk analysis of fxSCA7 92Q transgenic mouse cerebella.[15,16,44] The panel of analyzed genes represents distinct aspects of a complex molecular cascade implicated in SCA7 pathogenesis. Calbindin, encoded by the *Calb1* gene, is a representative of calcium regulatory genes involved in maintaining proper calcium homeostasis in cells. The downregulation of *Calb1* and other calcium flux genes, and the resultant calcium-dependent dysfunction of neuronal electrophysiology, have been linked to a reduced level of SIRT1 transactivation activity.[44]

Importin-5 is a protein mediating the bulk import of cargo to the nucleus and has been mechanistically linked to disrupted nuclear transport pathways in SCA1.[49] The fact that importin-5 also mediates the nuclear transport of H3 and H4 histones hints at a possible involvement of this factor in the epigenetic dysregulation observed in SCA7 mice.[50]

Aldoc loss and consequent zebrin-II type PC degeneration have recently been proposed by us to be a unifying pathomechanism across polyglutamine ataxias as loss of striped zebrin-II expression is observed in mouse models of SCA1, SCA2, SCA3, and SCA7.[16] Additionally, *Fam107b* expression has been shown to be downregulated in SCA1, 2, and 7 cerebella.[15] Although more research is necessary to assess the precise role of this factor in the ataxic phenotype, the indication that its downregulation occurs prior to the onset of disease

symptoms suggests a potentially causative role for altered expression of *Fam107b*, rather than a compensatory effect.

In this study, we also examined the expression of selected cerebellum-enriched PDE superfamily members in PCs from SCA7 and WT animals. PDEs are key components in the cyclic nucleotide signaling pathway, which controls various physiological processes in the brain, including protein kinase cascades, neurogenesis, synaptic transmission, and neuronal plasticity.[51] In the context of HD, a related polyglutamine disorder, one of the earliest gene expression signatures impacting patients is the transcriptional dysregulation of the cyclic nucleotide signaling pathway.[52] Moreover, inhibiting PDEs appears to be a promising strategy for developing new treatments for neurodegenerative disorders.[53] The reduced levels of *Pde10a* that we discovered in SCA7 PC nuclei have been observed in various cell and mouse models of HD, as well as in the brains of individuals with HD and PD.[21,52,54–56] Interestingly, despite decreased *Pde10a* expression, PDE10a inhibition can still rescue the neuromotor phenotype in HD mice in a CREB-dependent manner, implying that PDE10a deficiency is a secondary adaptation to counteract disrupted cyclic nucleotide signaling.[57] More research is needed to determine whether *Pde10a* regulation and activity in SCA7 are mechanistically related to the pathway changes observed in HD and PD.

Our data also revealed that *Pde9a* expression was significantly decreased and *Pde1c* expression was significantly increased in SCA7 PCs. While there is no existing evidence of *Pde9a* expression changes in degenerating brains, prior studies have shown that inhibition of PDE9a can improve memory and increase synaptic plasticity in healthy rodents and AD models, as well as protect against the toxic effects of β-amyloid.[58,59] Similarly, *Pde1c* transcription upregulation has been reported in mouse and human failing hearts, and genetic knockout of *Pde1c* can attenuate pathological cardiac remodeling and dysfunction both *in vitro* and *in vivo*.[60] Overall, our findings indicate that disruption of the cyclic nucleotide signaling pathway may play a role in SCA7 disease pathogenesis, suggesting that further research is warranted to determine whether PDE inhibition could be used as a therapeutic strategy for SCA7.

Limitations of the study

One notable limitation of gene expression analysis using nuclei as input is the difference in transcriptomic profiles between whole-cell and nuclear fractions. While employing nuclei has become a common practice in single-cell omics of neuronal tissue, the spatial distribution of mRNA molecules throughout the entire neuronal cell, particularly their significant presence in dendrites, presents a challenge in fully comprehending gene expression regulation in these cells.[61] We recognize this limitation as the next significant challenge in understanding cell-specific pathologies in neurodegenerative diseases, which may be addressed with the advancement of high-throughput and high-resolution spatial transcriptomic methods.

STAR★METHODS

Detailed methods are provided in the online version of this paper and include the following:

- KEY RESOURCES TABLE
- RESOURCE AVAILABILITY
  - Lead contact
  - Materials availability
  - Data and code availability
- EXPERIMENTAL MODEL AND STUDY PARTICIPANT DETAILS
  - Animals
- METHOD DETAILS
  - Buffers
  - Tissue preparation and nuclear extraction
  - Nuclei sorting
  - Nucleoli staining
  - Imaging flow cytometry
  - RNA isolation and gene expression analysis
  - Immunohistochemistry
  - Western blotting
- QUANTIFICATION AND STATISTICAL ANALYSIS

SUPPLEMENTAL INFORMATION

Supplemental information can be found online at https://doi.org/10.1016/j.crmeth.2024.100816.

ACKNOWLEDGMENTS

This work was supported by grants from both NIH (EY014061 and NS122140, to A.R.L.S. and R35HG011332 to C.B.L.) and from the National Science Centre, Poland (Sonata17 - UMO-2021/43/D/NZ3/03006). The flow cytometry data were acquired using the infrastructure developed under the project NEBI - National Imaging Centre for Biological and Biomedical Sciences, POIR.04.02.00-00-C004/19, co-financed through the European Regional Development Fund in the frame of Smart Growth Operational Programme 2014–2020 (Measure 4.2, Development of modern research infrastructure of the science sector).

AUTHOR CONTRIBUTIONS

P.M.S., A.R.L.S., and L.C.B. provided the conceptual framework for the study. P.M.S., A.R.L.S., L.C.B., M.F., and G.A. designed the experiments. L.C.B., M.F., G.A., A.F., A.R.L.S., and P.M.S. performed the experiments. M.T., A.S.-C., and P.J. performed flow cytometry sorting and analysis of mouse cerebella. P.M.S., A.R.L.S., and L.C.B. wrote the manuscript.

DECLARATION OF INTERESTS

The authors declare no competing interests.

Received: December 19, 2023
Revised: May 8, 2024
Accepted: June 17, 2024
Published: July 8, 2024

REFERENCES

1. Cerminara, N.L., Lang, E.J., Sillitoe, R.V., and Apps, R. (2015). Redefining the cerebellar cortex as an assembly of non-uniform Purkinje cell microcircuits. Nat. Rev. Neurosci. *16*, 79–93.
2. De Zeeuw, C.I., and Ten Brinke, M.M. (2015). Motor Learning and the Cerebellum. Cold Spring Harb. Perspect. Biol. *7*, a021683.
3. Cook, A.A., Fields, E., and Watt, A.J. (2021). Losing the Beat: Contribution of Purkinje Cell Firing Dysfunction to Disease, and Its Reversal. Neuroscience *462*, 247–261.
4. Davis, S., Scott, C., Ansorge, O., and Fischer, R. (2019). Development of a Sensitive, Scalable Method for Spatial, Cell-Type-Resolved Proteomics of the Human Brain. J. Proteome Res. *18*, 1787–1795.

5. Tomomura, M., Rice, D.S., Morgan, J.I., and Yuzaki, M. (2001). Purification of Purkinje cells by fluorescence-activated cell sorting from transgenic mice that express green fluorescent protein. Eur. J. Neurosci. *14*, 57–63.
6. Bouslama-Oueghlani, L., Wehrlé, R., Doulazmi, M., Chen, X.R., Jaudon, F., Lemaigre-Dubreuil, Y., Rivals, I., Sotelo, C., and Dusart, I. (2012). Purkinje Cell Maturation Participates in the Control of Oligodendrocyte Differentiation: Role of Sonic Hedgehog and Vitronectin. PloS one *7*, e49015. https://doi.org/10.1371/journal.pone.0049015.
7. Beguin, P., Kaneko, M., Chimura, T., and Suzuki, A.M. (2014). Digital expression profiling of the compartmentalized translatome of Purkinje neurons. Genome *24*, 1396–1410.
8. Mo, A., Mukamel, E.A., Davis, F.P., Luo, C., Henry, G.L., Picard, S., Urich, M.A., Nery, J.R., Sejnowski, T.J., Lister, R., et al. (2015). Epigenomic Signatures of Neuronal Diversity in the Mammalian Brain. Neuron *86*, 1369–1384.
9. Kozareva, V., Martin, C., Osorno, T., Rudolph, S., Guo, C., Vanderburg, C., Nadaf, N., Regev, A., Regehr, W.G., and Macosko, E. (2021). A transcriptomic atlas of mouse cerebellar cortex comprehensively defines cell types. Nature *598*, 214–219.
10. Solovei, I., Grandi, N., Knoth, R., Volk, B., and Cremer, T. (2004). Positional changes of pericentromeric heterochromatin and nucleoli in postmitotic Purkinje cells during murine cerebellum development. Cytogenet. Genome Res. *105*, 302–310.
11. Lein, E.S., Hawrylycz, M.J., Ao, N., Ayres, M., Bensinger, A., Bernard, A., Boe, A.F., Boguski, M.S., Brockway, K.S., Byrnes, E.J., et al. (2007). Genome-wide atlas of gene expression in the adult mouse brain. Nature *445*, 168–176.
12. Uhlén, M., Fagerberg, L., Hallström, B.M., Lindskog, C., Oksvold, P., Mardinoglu, A., Sivertsson, Å., Kampf, C., Sjöstedt, E., Asplund, A., et al. (2015). Proteomics. Tissue-based map of the human proteome. Science *347*, 1260419.
13. Yoo, S.Y., Pennesi, M.E., Weeber, E.J., Xu, B., Atkinson, R., Chen, S., Armstrong, D.L., Wu, S.M., Sweatt, J.D., and Zoghbi, H.Y. (2003). SCA7 knockin mice model human SCA7 and reveal gradual accumulation of mutant ataxin-7 in neurons and abnormalities in short-term plasticity. Neuron *37*, 383–401.
14. Stoyas, C.A., Bushart, D.D., Switonski, P.M., Ward, J.M., Alaghatta, A., Tang, M.-B., Niu, C., Wadhwa, M., Huang, H., Savchenko, A., et al. (2020). Nicotinamide pathway-dependent Sirt1 activation restores calcium homeostasis to achieve neuroprotection in spinocerebellar ataxia type 7. Neuron *105*, 630–644.e9.
15. Niewiadomska-Cimicka, A., Doussau, F., Perot, J.-B., Roux, M.J., Keime, C., Hache, A., Piguet, F., Novati, A., Weber, C., Yalcin, B., et al. (2021). SCA7 Mouse Cerebellar Pathology Reveals Preferential Downregulation of Key Purkinje Cell-Identity Genes and Shared Disease Signature with SCA1 and SCA2. J. Neurosci. *41*, 4910–4936.
16. Bartelt, L.C., Switonski, P.M., Adamek, G., Carvalho, J., Duvick, L.A., Jarrah, S.I., McLoughlin, H.S., Scoles, D.R., Pulst, S.M., Orr, H.T., et al. (2023). Purkinje-Enriched snRNA-seq in SCA7 Cerebellum Reveals Zebrin Identity Loss as a Central Feature of Polyglutamine Ataxias. Preprint at bioRxiv. https://doi.org/10.1101/2023.03.19.533345.
17. Pilarzyk, K., Porcher, L., Capell, W.R., Burbano, S.D., Davis, J., Fisher, J.L., Gorny, N., Petrolle, S., and Kelly, M.P. (2022). Conserved age-related increases in hippocampal PDE11A4 cause unexpected proteinopathies and cognitive decline of social associative memories. Aging Cell *21*, e13687.
18. Kawamoto, E.M., Vasconcelos, A.R., Degaspari, S., Böhmer, A.E., Scavone, C., and Marcourakis, T. (2013). Age-related changes in nitric oxide activity, cyclic GMP, and TBARS levels in platelets and erythrocytes reflect the oxidative status in central nervous system. Age *35*, 331–342.
19. Hesse, R., Lausser, L., Gummert, P., Schmid, F., Wahler, A., Schnack, C., Kroker, K.S., Otto, M., Tumani, H., Kestler, H.A., et al. (2017). Reduced cGMP levels in CSF of AD patients correlate with severity of dementia and current depression. Alzheimer's Res. Ther. *9*, 17.
20. McLachlan, C.S., Chen, M.L., Lynex, C.N., Goh, D.L.M., Brenner, S., and Tay, S.K.H. (2007). Changes in PDE4D isoforms in the hippocampus of a patient with advanced Alzheimer disease. Arch. Neurol. *64*, 456–457.
21. Niccolini, F., Foltynie, T., Reis Marques, T., Muhlert, N., Tziortzi, A.C., Searle, G.E., Natesan, S., Kapur, S., Rabiner, E.A., Gunn, R.N., et al. (2015). Loss of phosphodiesterase 10A expression is associated with progression and severity in Parkinson's disease. Brain *138*, 3003–3015.
22. Giampà, C., Laurenti, D., Anzilotti, S., Bernardi, G., Menniti, F.S., and Fusco, F.R. (2010). Inhibition of the striatal specific phosphodiesterase PDE10A ameliorates striatal and cortical pathology in R6/2 mouse model of Huntington's disease. PLoS One *5*, e13417.
23. Gines, S., Seong, I.S., Fossale, E., Ivanova, E., Trettel, F., Gusella, J.F., Wheeler, V.C., Persichetti, F., and MacDonald, M.E. (2003). Specific progressive cAMP reduction implicates energy deficit in presymptomatic Huntington's disease knock-in mice. Hum. Mol. Genet. *12*, 497–508.
24. Paulson, H.L., Shakkottai, V.G., Clark, H.B., and Orr, H.T. (2017). Polyglutamine spinocerebellar ataxias - from genes to potential treatments. Nat. Rev. Neurosci. *18*, 613–626.
25. Renthal, W., Tochitsky, I., Yang, L., Cheng, Y.-C., Li, E., Kawaguchi, R., Geschwind, D.H., and Woolf, C.J. (2020). Transcriptional Reprogramming of Distinct Peripheral Sensory Neuron Subtypes after Axonal Injury. Neuron *108*, 128–144.e9.
26. Yang, L., Xu, M., Bhuiyan, S.A., Li, J., Zhao, J., Cohrs, R.J., Susterich, J.T., Signorelli, S., Green, U., Stone, J.R., et al. (2022). Human and mouse trigeminal ganglia cell atlas implicates multiple cell types in migraine. Neuron *110*, 1806–1821.e8.
27. Zhen, Y., Cullen, C.L., Ricci, R., Summers, B.S., Rehman, S., Ahmed, Z.M., Foster, A.Y., Emery, B., Gasperini, R., and Young, K.M. (2022). Protocadherin 15 suppresses oligodendrocyte progenitor cell proliferation and promotes motility through distinct signalling pathways. Commun. Biol. *5*, 511.
28. Gallegos, D.A., Minto, M., Liu, F., Hazlett, M.F., Aryana Yousefzadeh, S., Bartelt, L.C., and West, A.E. (2023). Cell-type specific transcriptional adaptations of nucleus accumbens interneurons to amphetamine. Mol. Psychiatry *28*, 3414–3428. https://doi.org/10.1038/s41380-022-01466-1.
29. Turrero García, M., Stegmann, S.K., Lacey, T.E., Reid, C.M., Hrvatin, S., Weinreb, C., Adam, M.A., Nagy, M.A., and Harwell, C.C. (2021). Transcriptional profiling of sequentially generated septal neuron fates. Elife *10*, e71545. https://doi.org/10.7554/eLife.71545.
30. Fernandez-Albert, J., Lipinski, M., Lopez-Cascales, M.T., Rowley, M.J., Martin-Gonzalez, A.M., Del Blanco, B., Corces, V.G., and Barco, A. (2019). Immediate and deferred epigenomic signatures of in vivo neuronal activation in mouse hippocampus. Nat. Neurosci. *22*, 1718–1730.
31. Green, M.V., Gallegos, D.A., Boua, J.-V., Bartelt, L.C., Narayanan, A., and West, A.E. (2023). Single-Nucleus Transcriptional Profiling of GAD2-Positive Neurons From Mouse Lateral Habenula Reveals Distinct Expression of Neurotransmission- and Depression-Related Genes. Biol. Psychiatry Glob. Open Sci. *3*, 686–697.
32. Chen, X., Du, Y., Broussard, G.J., Kislin, M., Yuede, C.M., Zhang, S., Dietmann, S., Gabel, H., Zhao, G., Wang, S.S.-H., et al. (2022). Transcriptomic mapping uncovers Purkinje neuron plasticity driving learning. Nature *605*, 722–727.
33. Barski, J.J., Dethleffsen, K., and Meyer, M. (2000). Cre recombinase expression in cerebellar Purkinje cells. Genesis *28*, 93–98.
34. Witter, L., Rudolph, S., Pressler, R.T., Lahlaf, S.I., and Regehr, W.G. (2016). Purkinje Cell Collaterals Enable Output Signals from the Cerebellar Cortex to Feed Back to Purkinje Cells and Interneurons. Neuron *91*, 312–319.
35. Zhang, X.-M., Ng, A.H.-L., Tanner, J.A., Wu, W.-T., Copeland, N.G., Jenkins, N.A., and Huang, J.-D. (2004). Highly restricted expression of Cre recombinase in cerebellar Purkinje cells. Genesis *40*, 45–51.
36. Song, A.J., and Palmiter, R.D. (2018). Detecting and Avoiding Problems When Using the Cre–lox System. Trends Genet. *34*, 333–340.

37. Goldberg, M.W. (2017). Nuclear pore complex tethers to the cytoskeleton. Semin. Cell Dev. Biol. *68*, 52–58.
38. Jahed, Z., Domkam, N., Ornowski, J., Yerima, G., and Mofrad, M.R.K. (2021). Molecular models of LINC complex assembly at the nuclear envelope. J. Cell Sci. *134*, jcs258194. https://doi.org/10.1242/jcs.258194.
39. Harvey, R.J., and Napper, R.M. (1991). Quantitative studies on the mammalian cerebellum. Prog. Neurobiol. *36*, 437–463.
40. Niewiadomska-Cimicka, A., Hache, A., and Trottier, Y. (2020). Gene Deregulation and Underlying Mechanisms in Spinocerebellar Ataxias With Polyglutamine Expansion. Front. Neurosci. *14*, 571.
41. Kumar, A., Vaish, M., and Ratan, R.R. (2014). Transcriptional dysregulation in Huntington's disease: a failure of adaptive transcriptional homeostasis. Drug Discov. Today *19*, 956–962.
42. Wang, X., Allen, M., Li, S., Quicksall, Z.S., Patel, T.A., Carnwath, T.P., Reddy, J.S., Carrasquillo, M.M., Lincoln, S.J., Nguyen, T.T., et al. (2020). Deciphering cellular transcriptional alterations in Alzheimer's disease brains. Mol. Neurodegener. *15*, 38.
43. Butti, Z., and Patten, S.A. (2018). RNA Dysregulation in Amyotrophic Lateral Sclerosis. Front. Genet. *9*, 712.
44. Stoyas, C.A., Bushart, D.D., Switonski, P.M., Ward, J.M., Alaghatta, A., Tang, M.-B., Niu, C., Wadhwa, M., Huang, H., Savchenko, A., et al. (2020). Nicotinamide Pathway-Dependent Sirt1 Activation Restores Calcium Homeostasis to Achieve Neuroprotection in Spinocerebellar Ataxia Type 7. Neuron *105*, 630–644.e9.
45. Helmlinger, D., Hardy, S., Sasorith, S., Klein, F., Robert, F., Weber, C., Miguet, L., Potier, N., Van-Dorsselaer, A., Wurtz, J.-M., et al. (2004). Ataxin-7 is a subunit of GCN5 histone acetyltransferase-containing complexes. Hum. Mol. Genet. *13*, 1257–1265.
46. Palhan, V.B., Chen, S., Peng, G.-H., Tjernberg, A., Gamper, A.M., Fan, Y., Chait, B.T., La Spada, A.R., and Roeder, R.G. (2005). Polyglutamine-expanded ataxin-7 inhibits STAGA histone acetyltransferase activity to produce retinal degeneration. Proceedings of the National Academy of Sciences *102*, 8472–8477. https://doi.org/10.1073/pnas.0503505102.
47. Niu, C., Prakash, T.P., Kim, A., Quach, J.L., Huryn, L.A., Yang, Y., Lopez, E., Jazayeri, A., Hung, G., Sopher, B.L., et al. (2018). Antisense oligonucleotides targeting mutant Ataxin-7 restore visual function in a mouse model of spinocerebellar ataxia type 7. Sci. Transl. Med. *10*, eaap8677. https://doi.org/10.1126/scitranslmed.aap8677.
48. Switonski, P.M., Delaney, J.R., Bartelt, L.C., Niu, C., Ramos-Zapatero, M., Spann, N.J., Alaghatta, A., Chen, T., Griffin, E.N., Bapat, J., et al. (2021). Altered H3 histone acetylation impairs high-fidelity DNA repair to promote cerebellar degeneration in spinocerebellar ataxia type 7. Cell Rep. *37*, 110062.
49. Zhang, S., Williamson, N.A., Duvick, L., Lee, A., Orr, H.T., Korlin-Downs, A., Yang, P., Mok, Y.-F., Jans, D.A., and Bogoyevitch, M.A. (2020). The ataxin-1 interactome reveals direct connection with multiple disrupted nuclear transport pathways. Nat. Commun. *11*, 3343.
50. Soniat, M., Cağatay, T., and Chook, Y.M. (2016). Recognition Elements in the Histone H3 and H4 Tails for Seven Different Importins. J. Biol. Chem. *291*, 21171–21183.
51. Kelly, M.P. (2018). Cyclic nucleotide signaling changes associated with normal aging and age-related diseases of the brain. Cell. Signal. *42*, 281–291.
52. Niccolini, F., Haider, S., Reis Marques, T., Muhlert, N., Tziortzi, A.C., Searle, G.E., Natesan, S., Piccini, P., Kapur, S., Rabiner, E.A., et al. (2015). Altered PDE10A expression detectable early before symptomatic onset in Huntington's disease. Brain *138*, 3016–3029.
53. Erro, R., Mencacci, N.E., and Bhatia, K.P. (2021). The Emerging Role of Phosphodiesterases in Movement Disorders. Mov. Disord. *36*, 2225–2243.
54. Hebb, A.L.O., Robertson, H.A., and Denovan-Wright, E.M. (2004). Striatal phosphodiesterase mRNA and protein levels are reduced in Huntington′s disease transgenic mice prior to the onset of motor symptoms. Neuroscience *123*, 967–981.
55. Hu, H., McCaw, E.A., Hebb, A.L.O., and Gomez, G.T. (2004). Mutant huntingtin affects the rate of transcription of striatum-specific isoforms of phosphodiesterase 10A. Eur. J. *20*, 3351–3363.
56. Pagano, G., Niccolini, F., Wilson, H., Yousaf, T., Khan, N.L., Martino, D., Plisson, C., Gunn, R.N., Rabiner, E.A., Piccini, P., et al. (2019). Comparison of phosphodiesterase 10A and dopamine transporter levels as markers of disease burden in early Parkinson's disease. Mov. Disord. *34*, 1505–1515.
57. Beaumont, V., Zhong, S., Lin, H., Xu, W., Bradaia, A., Steidl, E., Gleyzes, M., Wadel, K., Buisson, B., Padovan-Neto, F.E., et al. (2016). Phosphodiesterase 10A Inhibition Improves Cortico-Basal Ganglia Function in Huntington's Disease Models. Neuron *92*, 1220–1237.
58. Cameron, R.T., Whiteley, E., Day, J.P., Parachikova, A.I., and Baillie, G.S. (2017). Selective inhibition of phosphodiesterases 4, 5 and 9 induces HSP20 phosphorylation and attenuates amyloid beta 1-42-mediated cytotoxicity. FEBS Open Bio *7*, 64–73.
59. Kroker, K.S., Mathis, C., Marti, A., Cassel, J.-C., Rosenbrock, H., and Dorner-Ciossek, C. (2014). PDE9A inhibition rescues amyloid beta-induced deficits in synaptic plasticity and cognition. Neurobiol. Aging *35*, 2072–2078.
60. Knight, W.E., Chen, S., Zhang, Y., Oikawa, M., Wu, M., Zhou, Q., Miller, C.L., Cai, Y., Mickelsen, D.M., Moravec, C., et al. (2016). PDE1C deficiency antagonizes pathological cardiac remodeling and dysfunction. Proc. Natl. Acad. Sci. USA *113*, E7116–E7125.
61. Perez, J.D., Dieck, S.T., Alvarez-Castelao, B., Tushev, G., Chan, I.C., and Schuman, E.M. (2021). Subcellular sequencing of single neurons reveals the dendritic transcriptome of GABAergic interneurons. Elife *10*, e63092. https://doi.org/10.7554/eLife.63092.

STAR★METHODS

KEY RESOURCES TABLE

REAGENT or RESOURCE	SOURCE	IDENTIFIER
Antibodies		
Mouse Monoclonal anti-Ran BP-2 Antibody (D-4)	Santa Cruz	Cat#: sc-74518; RRID: AB_2176784
Rabbit Monoclonal anti-Calbindin Antibody (D1I4Q)	Cell Signaling	Cat#: 13176; RRID: AB_2687400
Mouse Monoclonal anti-Myc Tag Antibody (9B11)	Cell Signaling	Cat#: 2276; RRID: AB_331783
Mouse Monoclonal anti-Histone H3 Antibody (1G1)	Santa Cruz	Cat#: sc-517576; RRID: AB_2848194
Mouse Monoclonal anti-GAPDH Antibody (6C5)	Thermo Fisher Scientific	Cat#: AM4300; RRID: AB_2536381
Rabbit anti-TOMM20 Antibody	Novus Biologicals	Cat#: NBP1-81556; RRID: AB_11003249
Goat anti-Mouse IgG (H + L), Recombinant Secondary Antibody, Alexa Fluor 488	Thermo Fisher Scientific	Cat#: A28175; RRID: AB_2536161
Goat anti-Rabbit IgG (H + L) Cross-Adsorbed Secondary Antibody, Alexa Fluor 594	Thermo Fisher Scientific	Cat#: A11012; RRID: AB_2534079
Peroxidase AffiniPure Donkey Anti-Rabbit IgG (H + L)	Jackson ImmunoResearch	Cat#: 711-035-152; RRID: AB_10015282
Peroxidase AffiniPure Donkey Anti-Mouse IgG (H + L)	Jackson ImmunoResearch	Cat#: 715-035-150; RRID: AB_2340770
Chemicals, peptides, and recombinant proteins		
Hoechst 33342	Thermo Fisher Scientific	Cat#: 62249
Sodium sulfate	Sigma-Aldrich	Cat#: 238597
Glucose	Sigma-Aldrich	Cat#: G7021
HEPES	Sigma-Aldrich	Cat#: H3375
Magnesium chloride	Chempur	Cat#: 116120500
Poly(1-vinylpyrrolidone-co-vinyl acetate)	Sigma-Aldrich	Cat#: 190845
Triton X-	BioShop Canada Inc.	Cat#: 9002-93-1
Bovine serum albumin	Cell Signaling	Cat#: 9998S
RNase inhibitor	Sigma-Aldrich	Cat#: R1158
Sodium sulfate	Sigma-Aldrich	Cat#: 238597
Nucleolus Bright Red	Dojindo	Cat#: N512-10
TRI Reagent	Thermo Fisher Scientific	Cat#: AM9738
Prolong Glass Antifade Mountant	Thermo Fisher Scientific	Cat#: P36980
Phenylmethylsulfonyl fluoride	Sigma-Aldrich	Cat#: 93482
Critical commercial assays		
Total RNA Zol Out -D kit	A&A Biotechnology	Cat#: 043-100
High-Capacity cDNA Reverse Transcription Kit	Thermo Fisher Scientific	Cat#: 4368814
TaqMan Fast Advanced Master Mix	Thermo Fisher Scientific	Cat#: 4444557
Calb1 TaqMan Assay	Thermo Fisher Scientific	Cat#: Mm00486647_m1
Ppp1r17 TaqMan Assay	Thermo Fisher Scientific	Cat#: Mm00495458_m1
Ryr1 TaqMan Assay	Thermo Fisher Scientific	Cat#: Mm01175211_m1
Pcp2 TaqMan Assay	Thermo Fisher Scientific	Cat#: Mm00435514_m1
Gabra6 TaqMan Assay	Thermo Fisher Scientific	Cat#: Mm01227754_m1
Aldoc TaqMan Assay	Thermo Fisher Scientific	Cat#: Mm01298116_g1
Ipo5 TaqMan Assay	Thermo Fisher Scientific	Cat#: Mm00659142_m1
Fam107b TaqMan Assay	Thermo Fisher Scientific	Cat#: Mm01325444_m1
Pde10a TaqMan Assay	Thermo Fisher Scientific	Cat#: Mm00449329_m1
Pde4d TaqMan Assay	Thermo Fisher Scientific	Cat#: Mm00456879_m1
Pde9a TaqMan Assay	Thermo Fisher Scientific	Cat#: Mm00501039_m1

(*Continued on next page*)

Continued		
REAGENT or RESOURCE	SOURCE	IDENTIFIER
Pde1c TaqMan Assay	Thermo Fisher Scientific	Cat#: Mm00478051_m1
Actb TaqMan Assay	Thermo Fisher Scientific	Cat#: Mm01205647_g1
Deposited data		
Original microscopy data	This paper	Zenodo: https://doi.org/10.5281/zenodo.11549380
Western blot uncropped data	This paper	Zenodo: https://doi.org/10.5281/zenodo.11549380
Experimental models: Organisms/strains		
Mouse: C57BL/6J	The Jackson Laboratory	Cat#: 000664, RRID:IMSR_JAX:000664
Mouse: B6.129-Gt(ROSA)26Sortm5.1(CAG-Sun1/sfGFP)Nat/MmbeJ	The Jackson Laboratory	Cat#: 030952, RRID:IMSR_JAX:030952
Mouse: B6.129-Tg(Pcp2-cre)2Mpin/J	The Jackson Laboratory	Cat#: 004146, RRID:IMSR_JAX:004146
Mouse: SCA7 266Q mice	Yoo et al.[13]	N/A
Software and algorithms		
GraphPad Prism 8.0	GraphPad	https://www.graphpad.com/scientific-software/prism/RRID: SCR_002798
FlowJo	FlowJo, LLC	https://www.flowjo.com/RRID: SCR_008520
IDEAS Image Analysis Software	Cytek Biosciences	https://cytekbio.com/pages/imagestream
Other		
50 mL polypropylene tubes	Sarstedt	Cat#: 62.548.004

RESOURCE AVAILABILITY

Lead contact

Further information and requests for resources and reagents should be directed to and will be fulfilled by the lead contact, Pawel Switonski (pswiton@ibch.poznan.pl).

Materials availability

This study did not generate new unique reagents.

Data and code availability

- Original microscopy data and uncropped western blot images reported in this paper have been deposited at Zenodo and are publicly available as of the date of publication. The DOI is listed in the key resources table. Flow cytometry data and Flow cytometry imaging data reported in this paper will be shared by the lead contactupon request.
- This paper does not report original code.
- Any additional information required to reanalyze the data reported in this paper is available from the lead contact upon request.

EXPERIMENTAL MODEL AND STUDY PARTICIPANT DETAILS

Animals

All animal experimentation adhered to NIH guidelines and was approved by Duke University and University of California, Irvine Institutional Animal Care and Use Committees. Wild type C57BL/6J (Strain #000664, RRID:IMSR_JAX:000664), CAG-Sun1/sfGFP reporter mice (Strain #030952, RRID:IMSR_JAX:030952) and PCP2-Cre line (Strain #004146, RRID:IMSR_JAX:004146) were obtained from Jackson Laboratories (Bar Harbor, Maine, USA). SCA7 heterozygous mice carrying 266 CAG repeats in the *Atxn7* gene, originally described by Yoo and colleagues,[13] have been maintained on the C57BL/6J strain background. Age-matched wild type littermates were used as controls. For all experiments both male and female mice were used with sex ratios equivalent to or not significantly different from 1:1. No significant differences in outcome measures between males and females were found. In the experiments involving SCA7 mice, only females were used because the breeding window for this specific line is limited and it was necessary to use males for reproduction (heterozygous females cannot reproduce). Mice were housed in groups of 2–5 per cage with a 12 h dark-light cycle and free access to food and water.

METHOD DETAILS

Buffers

Nucleus isolation protocol is based on the protocol published by Macosko laboratory,[9] with modifications. The following buffers were used in the experiments:

Dissociation buffer

82 mM Na2SO4 (238597, Sigma-Aldrich, St. Louis, Missouri, USA), 10 mM glucose (G7021, Sigma-Aldrich), 10 mM HEPES (H3375, Sigma-Aldrich), 5 mM MgCl2 (116120500, Chempur, Piekary Slaskie, Poland).

Extraction buffer

82 mM Na2SO4 (238597, Sigma-Aldrich), 10 mM glucose (G7021, Sigma-Aldrich), 10 mM HEPES (H3375, Sigma-Aldrich), 5 mM MgCl2 (116120500, Chempur), 1% (10 mg/mL) Poly(1-vinylpyrrolidone-co-vinyl acetate) (190845, Sigma-Aldrich), 1% Triton X-(9002-93-1, BioShop Canada Inc., Burlington, Canada), 0.01% BSA (9998S, Cell Signaling Technology, Danvers, Massachusetts, USA), 200–400 U/ml RNase inhibitor (R1158, Sigma-Aldrich).

Wash buffer

82 mM Na2SO4 (238597, Sigma-Aldrich), 10 mM glucose (G7021, Sigma-Aldrich), 10 mM HEPES (H3375, Sigma-Aldrich), 5 mM MgCl2 (116120500, Chempur), 0.01% BSA (Cell Signaling Technology, 9998S), 6.6–13.2 U/ml RNase inhibitor (R1158, Sigma-Aldrich).

2x hoechst staining solution

4 μM Hoechst 33342 (62249, Thermo Fisher Scientific Inc., Waltham, Massachusetts, USA) in Wash Buffer.

Tissue preparation and nuclear extraction

Mouse cerebella were collected, divided into two equal parts, snap frozen in the liquid nitrogen and stored in −80°C until needed. On the day of preparation, a 24-well tissue culture plate and 50 mL conical tubes (1 per sample) were coated for 1 h on ice with 1% BSA in Dissociation Buffer. We utilized 50 mL polypropylene tubes (62.548.004 Sarstedt, Numbrecht, Germany) since we observed that not all 50 mL conical tubes effectively pellet the nuclei post-centrifugation. All subsequent steps were performed on ice. Cerebellar halves were transferred from −80°C into a well of a 24-well plate containing 1 mL of Extraction Buffer and triturated by pipetting up and down 20 times with a 5 mL wide-bore tip and four rounds of 15 times with a regular 1 mL tip. Following dissociation, samples appeared cloudy with small chunks of unbroken tissue. Next, the entire sample volume was passed twice through a 26-gauge needle and filtered into a 50 mL conical tube through a 40 μM cell strainer. Filters were rinsed with 900 μL of 2x Hoechst Staining Solution and samples were incubated for 1 min on ice. Samples were filled with 30 mL of ice-cold Wash Buffer and centrifuged at 600 g at 4°C for 10 min using a swing bucket rotor. Subsequently, the supernatant was decanted and the pelleted nuclei were resuspended in 360 μL of Dissociation Buffer containing 5% BSA for blocking. Nuclei were incubated for 30 min at 4°C using a horizontal shaker. After blocking, 3.6 μL of Alexa Fluor 488 labeled anti-RanBP2 (D-4) antibody (sc-74518, Santa Cruz Biotechnology, Dallas, Texas, USA) was spiked into each sample (1:100), and nuclei were incubated for another 30 min at 4C. Next, 30 mL of Wash Buffer was added, and samples were centrifuged at 600g for 15 min at 4°C using a swing bucket rotor. After decanting supernatant and completely draining samples on kimwipes, nuclei were resuspended in 180 μL of Wash Buffer and stored on ice until sorted.

Nuclei sorting

Isolated nuclei were filtered through a 30 μm cell strainer and the filtrate was supplemented with 200 μL of 1% BSA in PBS right before being loaded into the sorter. Sorting was done on a high-speed flow cytometer BD FACSAria Fusion (Becton Dickinson, New Jersey, USA), using a 70 μm nozzle (70 psi) and a 1.5 ND filter on forward scatter (FSC), run with BD FACSFlow sheath fluid at room temperature, in a 4-way purity mode. Hoechst 33342 (Ex/Em 381/445 nm) was excited with 405 nm laser and detected in DAPI channel (450 BP/40), GFP (Ex/Em 398 and 475/509 nm) and Alexa Fluor 488 were excited with 488 nm laser and detected in FITC channel (530 BP/30). Data were analyzed using FACSDiva 9.0.1 software (Becton Dickinson) and FlowJo 10.8.1 (Becton Dickinson). FSC was plotted in linear scale, while SSC and fluorescence in logarithmic scale to facilitate gating relying on strong side scatter and respective fluorochrome signals.

Nucleoli staining

The protocol for staining nuclei envelopes was modified by adding Nucleolus Bright Red dye (N512-10, Dojindo, Japan) to the samples at a concentration of 5 μM after the nuclei were filtered through a 40 μm cell strainer. The samples were then incubated for 10 min on ice and further stained with Hoechst 33342 at a concentration of 2 μM for another 2 min. Afterward, the samples were washed with 30 mL of ice-cold wash buffer, centrifuged at 600g for 10 min using a swing bucket rotor, and then stained with anti-RanBP2 (Santa Cruz Biotechnology). The nucleoli were examined on the ImageStreamX Mark II flow cytometer (Luminex, Seattle, Washington, USA).

Imaging flow cytometry

Imaging flow cytometry was performed on an ImageStreamX Mark II cytometer equipped with two CCD camera detectors (Luminex). INSPIRE v201.1.0.765 software (Luminex) was used for data acquisition. The images were captured using a 60x objective at a low fluidics speed. For the analysis of sorted samples, brightfield was collected in channels 4 and 10, side scatter (SSC) was collected in

channel 6 (745–785 nm filter), at a 785 nm laser power of 3.44 mW. Hoechst 33342 was detected in channel 7 (480–560 nm filter) at a 405 nm laser power of 300 mW.

For nucleoli visualization and counting, brightfield was collected in channels 1 and 9, side scatter (SSC) was collected in channel 6 (745–785 nm filter), at a 785 nm laser power of 3.44 mW. Hoechst 33342 was detected in channel 7 (480–560 nm filter) at a 405 nm laser power of 40 mW, GFP and Alexa Fluor 488 were detected in channel 2 (480–560 nm filter) at a 488 nm laser power of 100/60 mW, respectively. Nucleolus Bright Red was detected in channel 4 (595–642 nm filter) at a 488 nm laser power of 100 mW (when used together with GFP) or 60 mW (when used together with Alexa Fluor 488). IDEAS v6.3 software (Luminex) was used for data visualization and analysis. In addition, for nucleoli counting, pictures collected in channels 4 and 7 were exported as TIF files.

RNA isolation and gene expression analysis

4,000 isolated nuclei were directly sorted into 100 μL of Tri Reagent (AM9738, Invitrogen, Waltham, Massachusetts, USA) and stored on ice. Total RNA was isolated using the Total RNA Zol Out -D kit (043–100, A&A Biotechnology, Poland) according to the manufacturer's instructions, with an elution volume of 30 μL. Subsequently, 10 μL of isolated RNA was reverse transcribed at 37°C for 120 min with the High-Capacity cDNA Reverse Transcription Kit (4368814, Thermo Fisher Scientific) and random primers. TaqMan Fast Advanced Master Mix (4444557, Thermo Fisher Scientific) and the CFX Connect Real-Time PCR System (Bio-Rad, Hercules, California, USA) was used to quantify gene expression. The following Taqman probes were utilized in this study: *Calb1* Mm00486647_m1, Ppp1r17 Mm00495458_m1, *Ryr1* Mm01175211_m1, *Pcp2* Mm00435514_m1, *Gabra6* Mm01227754_m1, *Aldoc* Mm01298116_g1, *Ipo5* Mm00659142_m1, *Fam107b* Mm01325444_m1, *Pde10a* Mm00449329_m1, *Pde4d* Mm00456879_m1, *Pde9a* Mm00501039_m1, *Pde1c* Mm00478051_m1, *Actb* Mm01205647_g1 by Thermo Fisher Scientific.

Immunohistochemistry

Sun1/sfGFP+, Pcp2-Cre- and Sun1/sfGFP+, Pcp2-Cre+ mice were perfused with PBS followed by 4% PFA. Brains were then removed and post-fixed in 4% PFA overnight, followed by cryopreservation through immersion in solutions of increasing sucrose concentration (10%, 20%, and 30% sucrose/PBS) over a period of 24 h each. Afterward, brains were mounted in OCT and frozen in a liquid nitrogen isopentane bath. 25μm sagittal slices were obtained using a Leica CM3050S Cryostat. The staining process began with incubating the slices in 4% PFA in PBS for 10 min, followed by 7 min incubation in 0.05% Triton X- in PBS and one washing step in PBS. Blocking was performed with 5% BSA in PBS at room temperature for 1 h. Next, the slices were stained overnight at 4°C with primary antibodies: rabbit anti-calbindin (1:400, Cat. No. 13176, Cell Signaling) and mouse anti-Myc-Tag (1:200 Cat. No. 2276, Cell Signaling). After another round of washing (three times in PBS for 10 min each), the slices were stained with secondary fluorescent goat anti-mouse and rabbit antibodies (1:400 each, Cat No. A28175 and A11012, Thermo Fisher Scientific). Finally, the slices were stained with Hoechst 33342 (1:10000, Cat No. 62249, Thermo Fisher Scientific) for 10 min in PBS, washed twice for 10 min each in PBS, and then mounted onto microscope slides using Prolong Glass Antifade Mountant (Cat. No. P36980, Thermo Fisher Scientific). Slides were imaged on a Zeiss 880 Inverted Confocal microscope.

Western blotting

Samples obtained at various stages of the isolation protocol were collected in a PBS buffer containing 2mM PMSF (Sigma, Cat. No. 93482). Samples underwent two freeze-thaw cycles in liquid nitrogen, and subsequently, one-fifth of the total volume of 5x concentrated lysis buffer was added, resulting in a final concentration of 60 mM TRIS-base, 2% SDS, 10% sucrose, and 2 mM PMSF. For the cerebellar tissue, freeze-thaw cycles were replaced with repetitive passage of the sample through an 18G needle in the lysis buffer. Samples were then sonicated using a Pico Bioruptor (Diagenode) for ten cycles of 30 s on and 30 s off at 4°C. Lysates were centrifuged and quantified using BCA. 10μg of total protein was loaded per well of a 10% SDS-PAGE gel and transferred onto a nitrocellulose membrane. The membrane was blocked in 5% BSA (Cat. No.9998, Cell Signaling) in Tris-buffered saline +0.1% Tween 20 (TBST) for 1 h at room temperature, followed by overnight incubation with primary antibodies at 4°C. The antibodies used were as follows: Histone H3 (Cat. No. sc517576, Santa Cruz), GAPDH (Cat. No. AM4300, Thermo Fisher Scientific), and TOM20 (Cat No. NBP1-81556, Novus). All primary antibodies were used at a 1:1000 dilution. Secondary HRP antibodies were anti-rabbit (Cat No. 711-035-152, Jackson ImmunoResearch) or anti-mouse (Cat No. 715-035-150, Jackson ImmunoResearch) and were used at a 1:3000 dilution.

QUANTIFICATION AND STATISTICAL ANALYSIS

Statistical analysis was conducted using Prism 8.0 (GraphPad). All statistical tests were two-tailed Student's t-tests, with a significance level (alpha) consistently set to 0.05. Asterisks denote the degree of statistical significance: $^{*}p < 0.05$, $^{**}p < 0.01$, $^{***}p < 0.001$, $^{****}p < 0.0001$. Error bars in bar graphs represent the standard error of the mean (SEM). Detailed information regarding each experiment, including the number of biological replicates, is provided in the legend for each figure.

Cell Reports Methods, Volume *4*

Supplemental information

Antibody-assisted selective isolation

of Purkinje cell nuclei

from mouse cerebellar tissue

Luke C. Bartelt, Mouad Fakhri, Grazyna Adamek, Magdalena Trybus, Anna Samelak-Czajka, Paulina Jackowiak, Agnieszka Fiszer, Craig B. Lowe, Albert R. La Spada, and Pawel M. Switonski

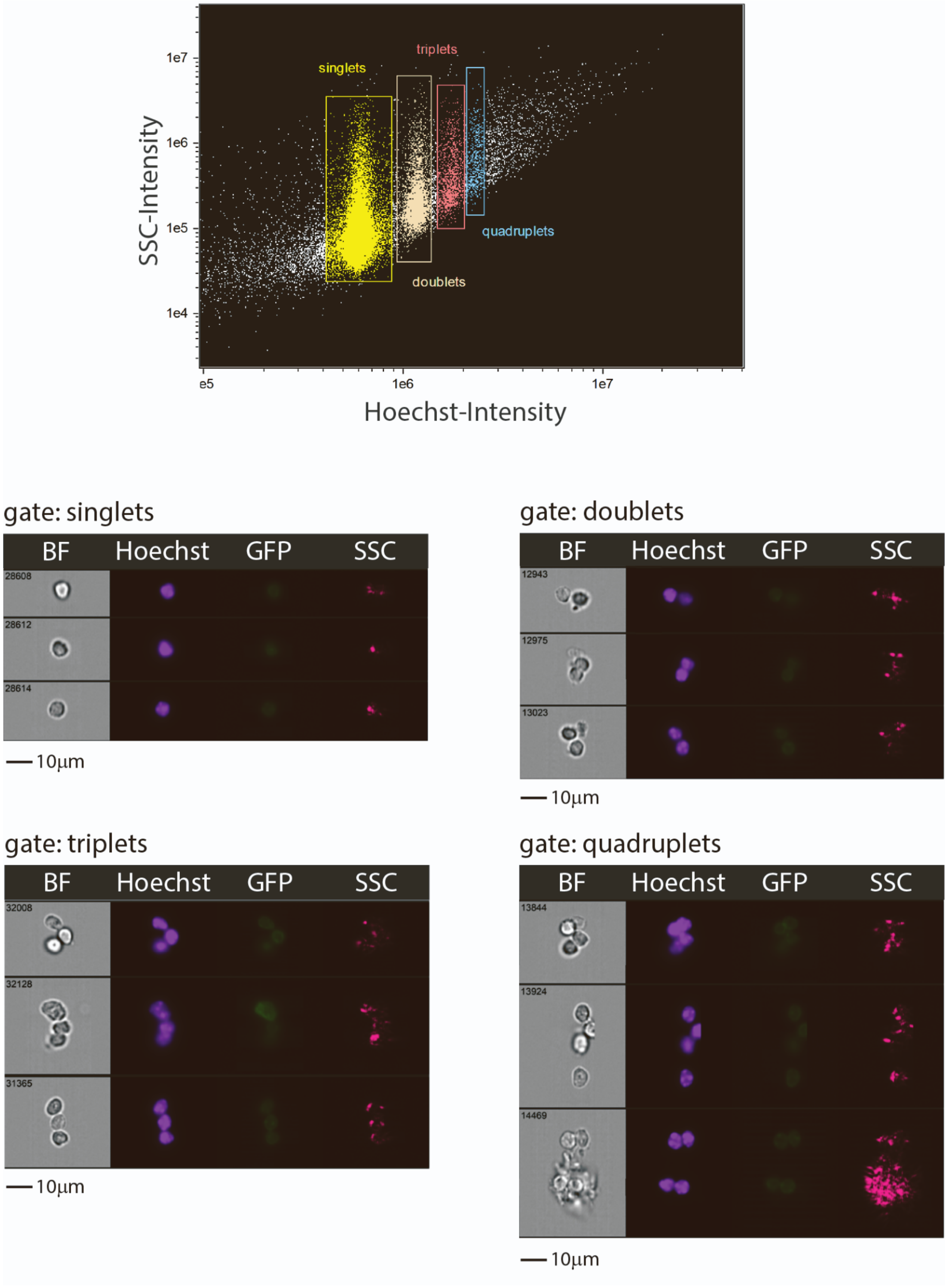

Supplementary Figure 1. Visual inspection of Hoechst staining patterns conducted on nuclei isolated from mouse cerebella, related to Figure 1.

Each cluster represents a gradual aggregation of nuclei, as verified through image cytometry. Representative examples are shown; Scale bar: 10µm. It is important to note that all nuclei sorted and analyzed in this study were selected from the singlets gate.

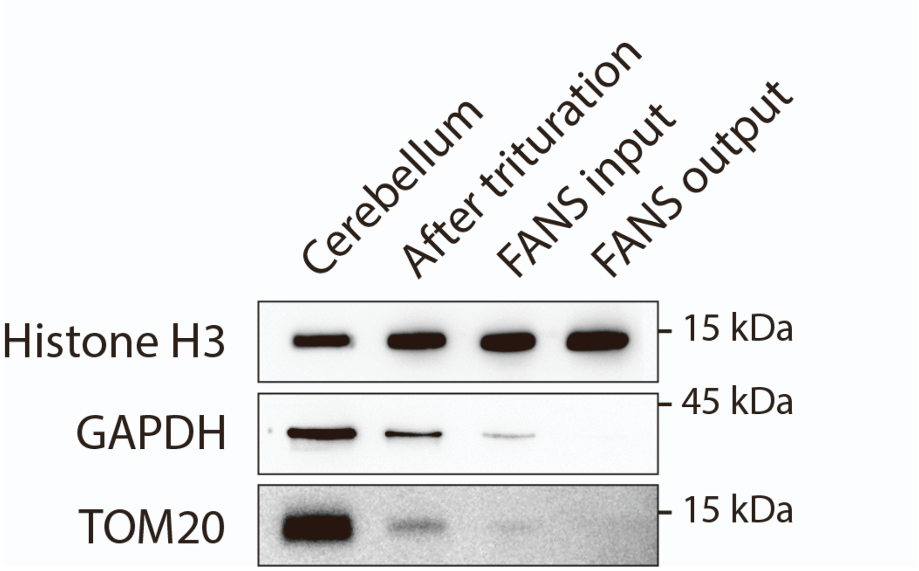

Supplementary Figure 2. The quality of nuclear fractionation assessed through Western blot analysis, related to the STAR Method - Western blotting.
Samples were obtained at various stages of the isolation protocol, including unlysed tissue, the fraction after trituration, the input fraction for FANS, and the output fraction from FANS. Equal protein amounts were loaded onto a gel for each fraction. The observations revealed a gradual decrease in cytosolic markers (GAPDH) and mitochondrial markers (TOM20) until they were no longer detectable in the FANS output fraction. Simultaneously, there was a gradual increase in the nuclear marker Histone H3, indicating the efficient removal of cytosolic components.

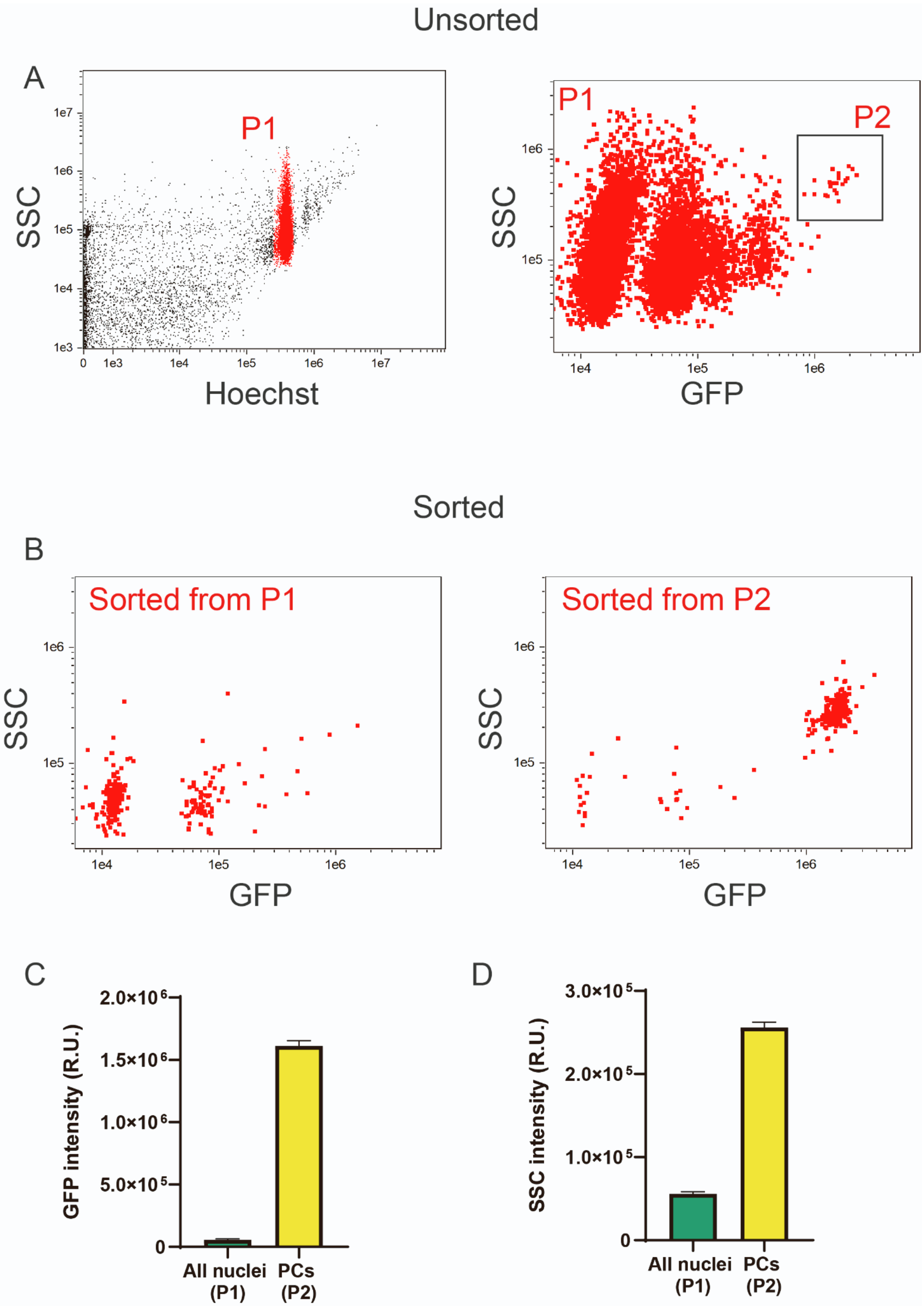

Supplementary Figure 3. ImageStream cytometric analysis of nuclei isolated from Sun1/sfGFP-positive and Pcp2-Cre-positive cerebella, related to Figure 2.
A) Both unsorted nuclei and B) nuclei previously sorted on the BD FACSAria Fusion flow cytometer were included in this analysis. C) The bar graphs illustrate variations in GFP intensity and D) side scatter (SSC) intensity, as computed by the IDEAS software, within the P1 and P2 populations indicated on the plots above.

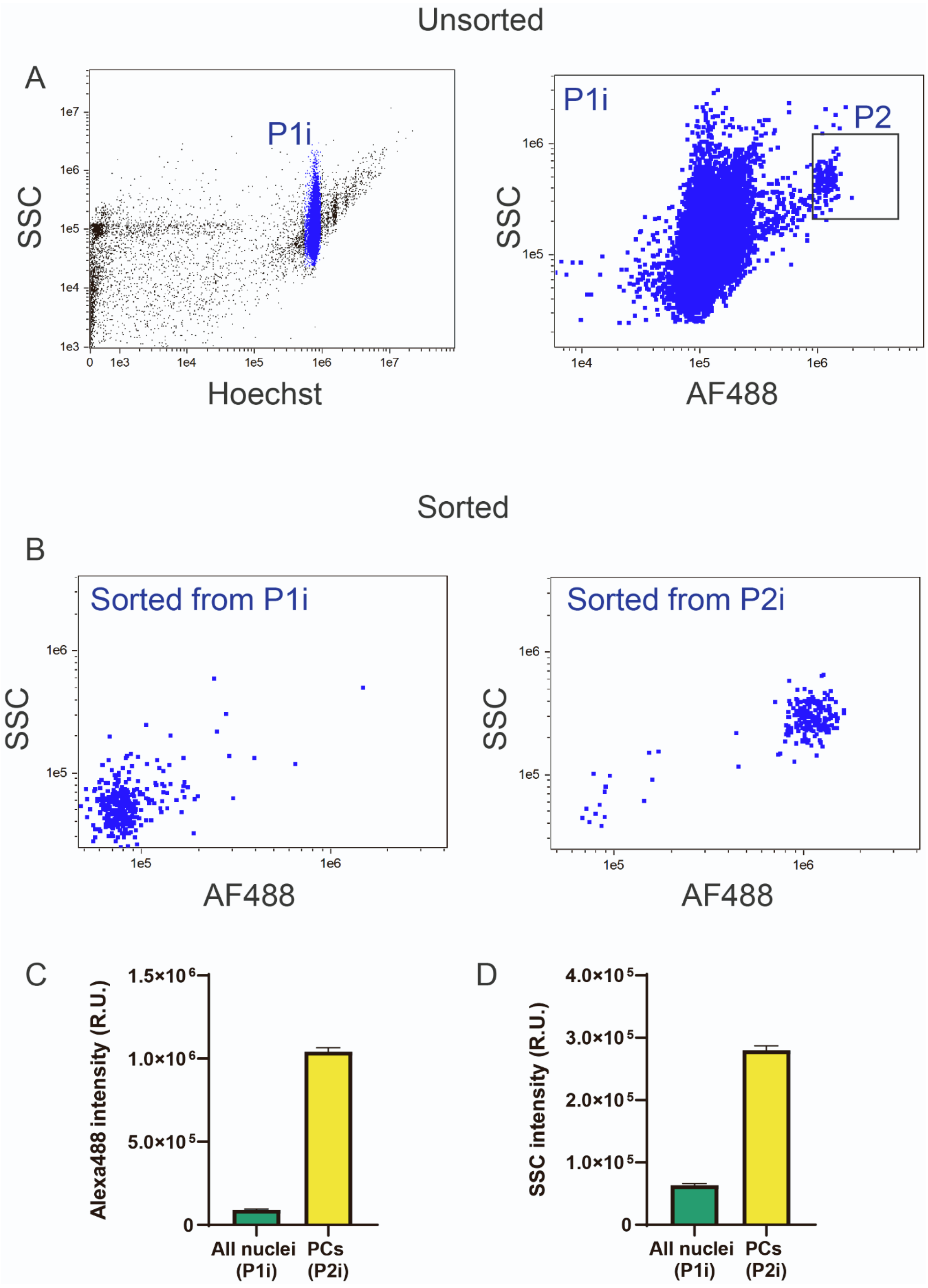

Supplementary Figure 4. ImageStream cytometric analysis performed on nuclei immunostained against RanBP2, which were isolated from wild-type cerebella, related to Figure 4.

A) Both unsorted nuclei and B) nuclei previously sorted on the BD FACSAria Fusion flow cytometer were included in this analysis. C) The bar graphs illustrate variations in AlexaFluor488 intensity and D) side scatter (SSC) intensity, as computed by the IDEAS software, within the P1i and P2i populations indicated on the plots above.

Supplementary Table 1: Contribution of PC Nuclei to Overall Singlet Nuclei (%), related to Figures 1 and 3.

% of PC in singlets	Sun1_GFP	RanBP2
exp_1	0.5	0.8
exp_2	0.4	1
exp_3	0.5	0.6
exp_4	0.4	0.9
exp_5	0.2	0.7
exp_6	0.3	0.6
exp_7	0.5	0.6
exp_8	0.3	0.5
exp_9	0.4	0.7
exp_10	0.3	0.3
exp_11	0.5	0.3
exp_12	0.3	0.7
exp_13	0.4	0.8
exp_14	0.5	0.5
exp_15	0.5	0.7
Average	0.40	0.65
